# Heterogeneous transfer learning model for improving the classification performance of fNIRS signals in motor imagery among cross-subject stroke patients

**DOI:** 10.3389/fnhum.2025.1555690

**Published:** 2025-03-27

**Authors:** Jin Feng, YunDe Li, ZiJun Huang, Yehang Chen, SenLiang Lu, RongLiang Hu, QingHui Hu, YuYao Chen, XiMiao Wang, Yong Fan, Jing He

**Affiliations:** ^1^Guilin Normal College, Student Affairs Office, Guilin, China; ^2^Guilin University of Aerospace Technology, Intelligent Detection and Information Processing Laboratory, Guilin, China; ^3^Guilin University of Electronic Technology, School of Electronic Engineering and Automation, Guilin, China; ^4^Department of Rehablitation Medicine, Jiangmen Central Hospital, Jiangmen, Guangdong, China; ^5^Guilin University of Aerospace Technology, School of Computer Science and Engineering, Guilin, China; ^6^Department of Medical Imaging, Nanxishan Hospital of Guangxi Zhuang Autonomous Region, Guilin, China; ^7^Jiangmen Key Laboratory of Artificial Intelligence in Medical Image Computation and Application, Jiangmen Central Hospital, Jiangmen, Guangdong, China; ^8^Office of the President, Guilin University of Aerospace Technology, Guilin, China; ^9^School of Management, Guilin University of Aerospace Technology, Guilin, China

**Keywords:** MI-fNIRS, Cross-Subject Heterogeneous Transfer Learning Model, Bayesian Extreme Learning Machine, stroke patients, BCI

## Abstract

**Introduction:**

Motor imagery functional near-infrared spectroscopy (MI-fNIRS) offers precise monitoring of neural activity in stroke rehabilitation, yet accurate cross-subject classification remains challenging due to limited training samples and significant inter-subject variability. This study proposes a Cross-Subject Heterogeneous Transfer Learning Model (CHTLM) to enhance the generalization of MI-fNIRS signal classification in stroke patients.

**Methods:**

CHTLM leverages labeled electroencephalogram (EEG) data from healthy individuals as the source domain. An adaptive feature matching network aligns task-relevant feature maps and convolutional layers between source (EEG) and target (fNIRS) domains. Multi-scale fNIRS features are extracted, and a sparse Bayesian extreme learning machine classifies the fused deep learning features.

**Results:**

Experiments utilized two MI-fNIRS datasets from eight stroke patients pre- and post-rehabilitation. CHTLM achieved average accuracies of 0.831 (pre-rehabilitation) and 0.913 (post-rehabilitation), with mean AUCs of 0.887 and 0.930, respectively. Compared to five baselines, CHTLM improved accuracy by 8.6–10.5% pre-rehabilitation and 11.3–15.7% post-rehabilitation.

**Discussion:**

The model demonstrates robust cross-subject generalization by transferring task-specific knowledge from heterogeneous EEG data while addressing domain discrepancies. Its performance gains post-rehabilitation suggest clinical potential for monitoring recovery progress. CHTLM advances MI-fNIRS-based brain-computer interfaces in stroke rehabilitation by mitigating data scarcity and variability challenges.

## Introduction

Stroke is one of the leading causes of adult disability, with 70–85% of first-time patients experiencing hemiplegia (Nutter, 2023). A major clinical challenge in stroke rehabilitation is the lack of objective, quantitative indicators for assessing motor function recovery. Traditional evaluation methods rely on subjective clinical assessments, which can lead to inconsistencies in diagnosis and treatment planning. As an innovative active rehabilitation approach, motor imagery brain-computer interface (MI-BCI) technology enables the collection of brain signals during motor imagery tasks, decodes brain intentions, and converts them into control commands for rehabilitation training devices. These devices assist patients in executing movements, thereby facilitating neural pathway remodeling and promoting the recovery of motor functions (Sebastián-Romagosa et al., 2020). This rehabilitation method holds great promise in clinical applications and offers significant therapeutic value (Vourvopoulos et al., 2019). Functional near-infrared spectroscopy (fNIRS) is an optical imaging technique that measures hemodynamic responses associated with neural activity by detecting changes in oxyhemoglobin (HbO) and deoxyhemoglobin (HbR) concentrations. This non-invasive method provides high spatial resolution and can effectively capture cortical activation patterns during motor imagery tasks.

MI has high temporal resolution but is susceptible to noise, while fNIRS offers better spatial resolution and signal stability but has lower temporal resolution, making it difficult to capture rapid brain activity changes. The integration of MI and fNIRS (MI-fNIRS) offers a promising approach for neurorehabilitation. The combination of both modalities effectively compensates for the limitations of each single modality, improving data quality and enriching feature information. MI-fNIRS enables real-time monitoring of cortical activation during imagined movements, making it a valuable tool for brain-computer interface (BCI) applications in stroke rehabilitation. In this study, we adopt the MI-fNIRS fusion approach and introduce the Cross-Subject Heterogeneous Transfer Learning Model (CHTLM) to further overcome inter-subject variability, improve the classification accuracy of MI-fNIRS signals, and better support stroke rehabilitation training.

## Related work

In recent years, convolutional neural networks (CNNs), renowned for their powerful feature-learning capabilities, have been extensively applied in the field of MI-BCI. They provide an effective approach for processing and analyzing brain signals (Khademi et al., 2023). Although CNNs exhibit great potential in MI-BCI applications, training high-performing models typically requires large amounts of data. However, in medical research, acquiring MI-fNIRS data from stroke patients necessitates specialized equipment, skilled professionals, and expert annotations from medical practitioners (Leamy, 2015). This process is both time-consuming and costly, often resulting in insufficient training samples for CNN-based MI-fNIRS classification tasks (Ma et al., 2021).

Additionally, inter-individual variability in brain signal characteristics poses a major challenge for cross-subject classification models. Models trained on data from one patient often fail to generalize effectively when applied to new patients, leading to poor generalization performance (Lee et al., 2024). Thus, applying CNNs to MI-fNIRS classification is hindered by both the limited availability of training data and substantial inter-subject variability.

To address these challenges, researchers have explored transfer learning (TL), which offers advantages in scenarios with limited training data (Salehi et al., 2023). TL facilitates knowledge transfer from a source domain to improve learning in a target domain, thereby enhancing model performance (Limpiti et al., 2021). In heterogeneous transfer learning applications within the MI-fNIRS domain, feature representation and distribution disparities exist between the source (EEG data) and target (fNIRS data) domains (Valverde et al., 2021). Consequently, heterogeneous transfer learning has gained attention (Bao et al., 2023), as it enables knowledge transfer between EEG and fNIRS—two distinct modalities with fundamentally different feature representations, data distributions, and signal characteristics (Day and Khoshgoftaar, 2017).

However, directly transferring all knowledge from the source domain can result in poor adaptation to the target task, leading to negative transfer effects that degrade model performance (Wang et al., 2019). Therefore, in MI-fNIRS tasks, effective knowledge selection and transfer mechanisms are crucial. It is essential to identify task-relevant knowledge and determine optimal integration points within the target model. Traditionally, researchers rely on manual methods to establish correspondences between source and target model layers, but this process is resource-intensive and lacks adaptability (Wang et al., 2020). Manual alignment may limit the efficiency of transfer learning, as it does not autonomously identify the optimal knowledge transfer locations.

This study introduces three key innovations: (1) The use of wavelet transformation to convert raw fNIRS signals into image data, enhancing clarity in displaying frequency components and temporal changes to enrich signal features. (2) The CHTLM algorithm employs an adaptive feature matching network to explore correlations between the source and target domains. It transfers useful knowledge from the source model relevant to the target task to appropriate positions in the target model, thereby enriching the target model’s knowledge and mitigating individual differences. (3) Integration of sparse Bayesian theory into the ELM algorithm to achieve sparse solutions, effectively alleviating overfitting issues and enhancing the model’s generalization capabilities.

## Materials

### Experimental paradigm

In the process of model development and validation, we primarily employed the motor imagery EEG data from the BCI Competition IV Dataset 2a (Mohammadi et al., 2022) as the source domain dataset, and collected fNIRS data from eight stroke patients performing motor imagery as the target domain dataset. Among them, fNIRS data belongs to the retrospective study data.

The source domain dataset comprises EEG data collected from eight subjects performing four different motor imagery (MI) tasks: left hand, right hand, foot, and tongue. The experimental design involved a trial process consisting of six runs, with each run containing 48 MI trials, resulting in a total of 288 trials—72 trials per task. Before the experiment, subjects were provided with sufficient rest and informed about the experimental procedure. At the beginning of each trial, a black screen with a fixed cross was displayed, followed by a short auditory cue. Two seconds later, an arrow pointing left, right, or in another direction appeared on the screen for 1.25 s, prompting the subject to perform the corresponding MI task. The task lasted for 6 s, after which the cross disappeared, signaling the subject to rest and prepare for the next trial.

**Figure 1:**
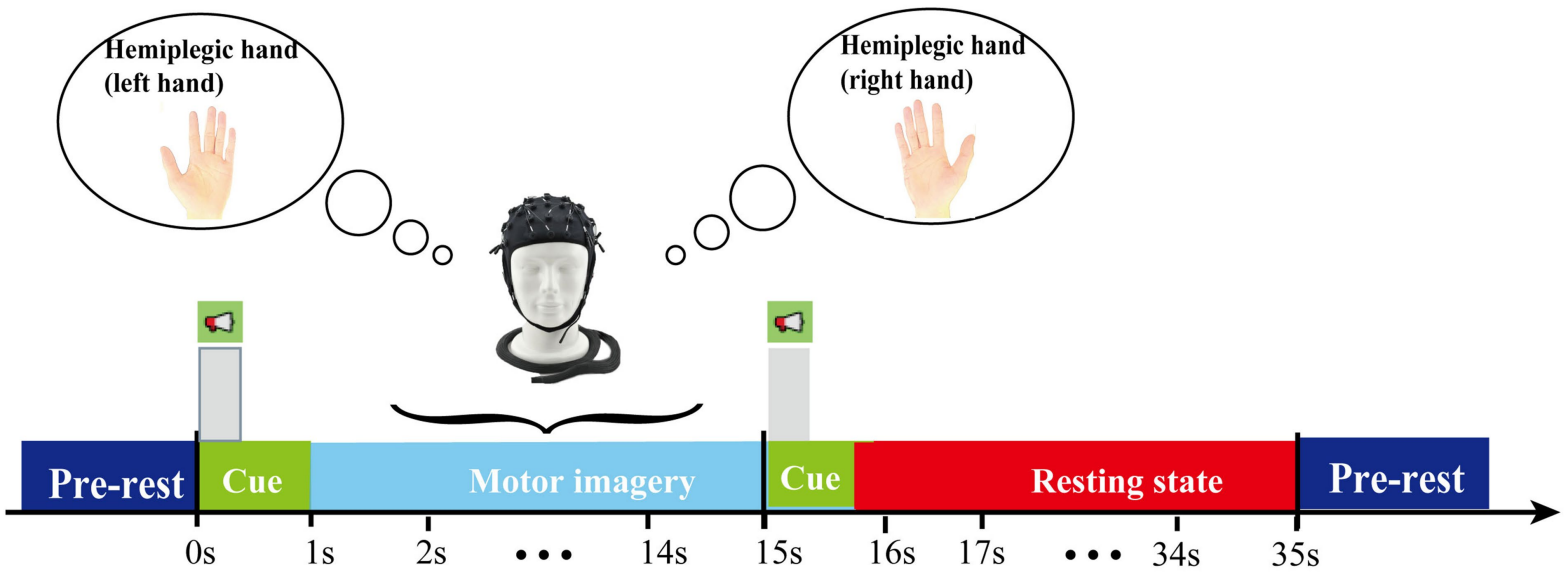
Timing scheme of the experimental paradigm.

The target domain dataset consists of fNIRS data collected from eight stroke patients performing MI tasks with their hemiplegic hand. This dataset is divided into two subsets: pre-rehabilitation and post-rehabilitation training data. During the experiment, participants underwent a trial process comprising 20 trials—10 MI task trials and 10 resting-state trials. The experimental paradigm is illustrated in Figure 1. Before the experiment, participants were allowed sufficient time to rest and were briefed on the procedure. The experiment commenced with a 10-s initial rest period, followed by a 1-s auditory cue. The participants then performed MI of their hemiplegic hand for 15 s. Another 1-s auditory cue was played, signaling the beginning of a 20-s rest period before the next trial. This process was repeated for 20 trials, concluding with a 3-s post-experiment rest period.

### Data acquisition

For the self-collected MI-fNIRS dataset, eight stroke patients aged 35 to 68 participated in the study, with an average age of 50.25 years and a sample variance of 57.76. All patients were fully conscious, able to understand instructions, and actively engaged in the rehabilitation process. They all exhibited hemiparesis due to either ischemic or hemorrhagic stroke. All participants voluntarily consented to the study and signed an informed consent form. Among them, four had left-hand hemiparesis, and four had right-hand hemiparesis.

**Figure 2:**
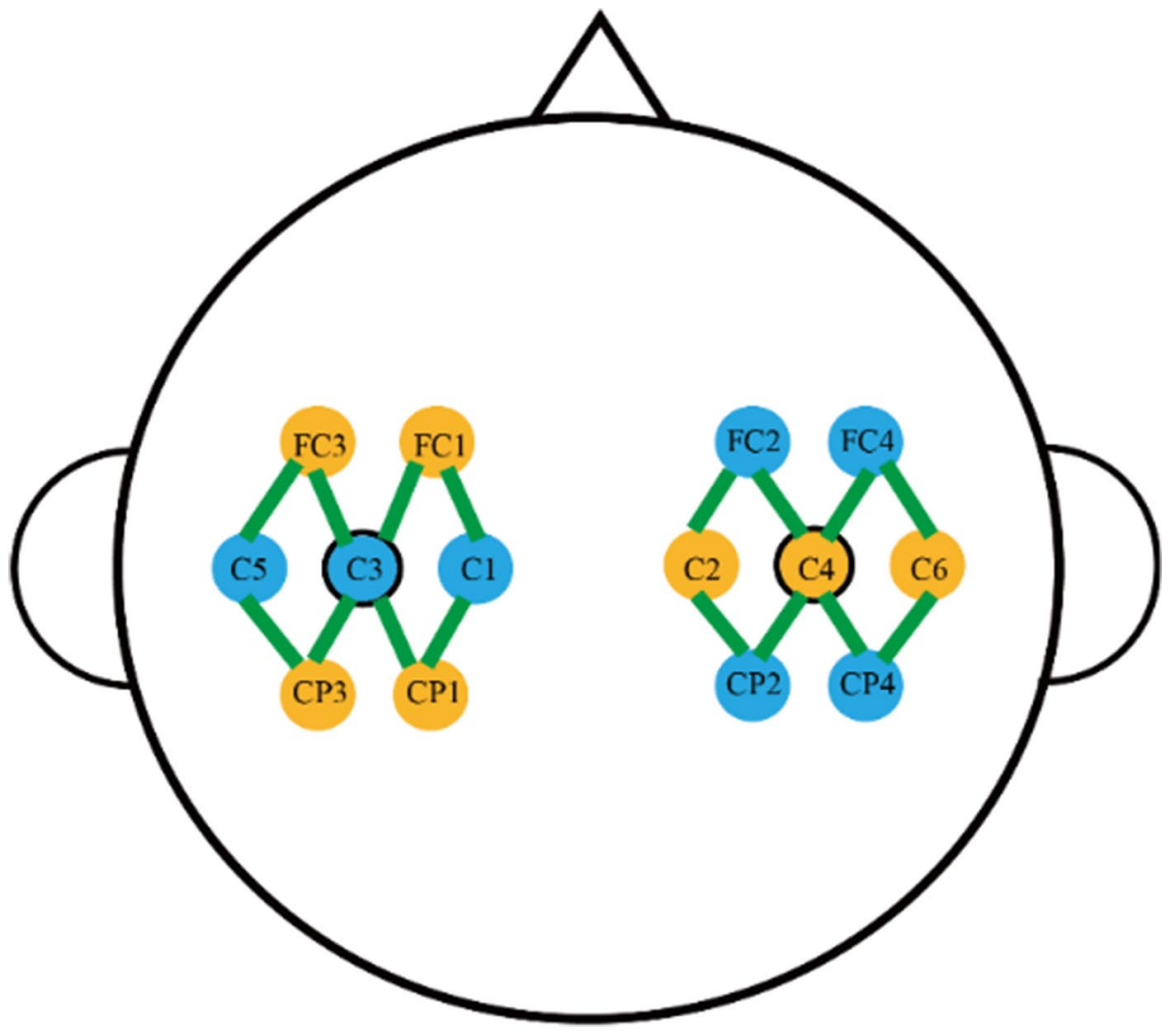
The positional distribution map of fNIRS optodes.

Data acquisition was conducted using the NirSmart 6000B medical-grade portable system, developed by Huichuang Medical Equipment Co., Ltd., Danyang, China. The system was equipped with seven light sources and seven detectors, forming a total of 16 channels, which were symmetrically placed on the motor cortex around C3 and C4, with eight channels per region. The source-detector distance was set to 30 mm, and data were collected at two wavelengths (730 nm and 850 nm) with a sampling rate of 11 Hz. The position distribution of the fNIRS optodes is shown in Figure 2. For details on the EEG signal acquisition of the source domain dataset, please refer to the Supplementary material S1.

Prior to rehabilitation training, patients’ brain fNIRS data were collected as pre-rehabilitation experimental data. They then underwent a two-week rehabilitation program. After completing the rehabilitation regimen, their fNIRS data were recorded again as post-rehabilitation experimental data. The rehabilitation intervention utilized a smart mechanical hand rehabilitation device that integrates pneumatic and electrical components. This device stimulates motor nerves through physiological electrical stimulation, regulating neural excitability in the hemiparetic hand, facilitating muscle contraction, and enhancing wrist and hand joint movement. Detailed specifications of the device are provided in Supplementary material S2.

## Methods

### Data preprocessing

To ensure the quality and experimental applicability of the self-collected motor imagery fNIRS data, we conducted a preprocessing procedure that primarily consists of five aspects: (1) Raw data conversion, where we applied the modified Beer–Lambert law to accurately convert the raw optical data into concentration changes of oxyhemoglobin (HbO_2_) and deoxyhemoglobin (HbR). (2) Channel selection, where we retained the HbO_2_ and HbR data from 16 channels in the motor cortex to focus on studying brain regions related to movement. (3) Filtering, where a bandpass filter of 0.01–0.2 Hz was applied to the initially processed data to effectively remove the interference of high-frequency noise and low-frequency baseline drift while retaining physiological signals closely related to changes in cerebral blood oxygen levels, thus improving the signal-to-noise ratio. (4) Time window processing, where, to align the data of motor imagery and resting states and ensure that the extracted cerebral blood oxygen responses are closely related to the experimental tasks, we utilized the fNIRS data corresponding to the 10s motor imagery task time and 10s resting state time in each trial. (5) Time-frequency representation of EEG signals based on STFT: EEG signals exhibit relatively stable rhythmic activity within well-defined frequency bands. STFT provides a fixed resolution across all frequencies, making it an ideal choice for extracting power spectral features within predefined frequency bands. Given the high temporal resolution of EEG (with a millisecond-level sampling rate), STFT leverages the fast Fourier transform (FFT) to achieve efficient spectral decomposition without excessive computational overhead. (6) Image-based time-frequency representation of fNIRS signals, considering that fNIRS signals have high spatial resolution and are two-dimensional time-series data containing multiple channels. Additionally, wavelet transform offers flexibility in processing time-series data (Yu et al., 2024). Unlike EEG signals, which exhibit distinct rhythmic oscillations in well-defined frequency bands, fNIRS signals contain slow hemodynamic variations that evolve across different time scales. Wavelet transform offers an adaptive time-frequency resolution, this makes it well-suited for capturing the complex temporal and spectral characteristics of fNIRS signals in the time-frequency domain. The details of the preprocessing steps for the source domain dataset can be found in Supplementary material S3.

### Construction of the CHTLM

**Figure 3:**
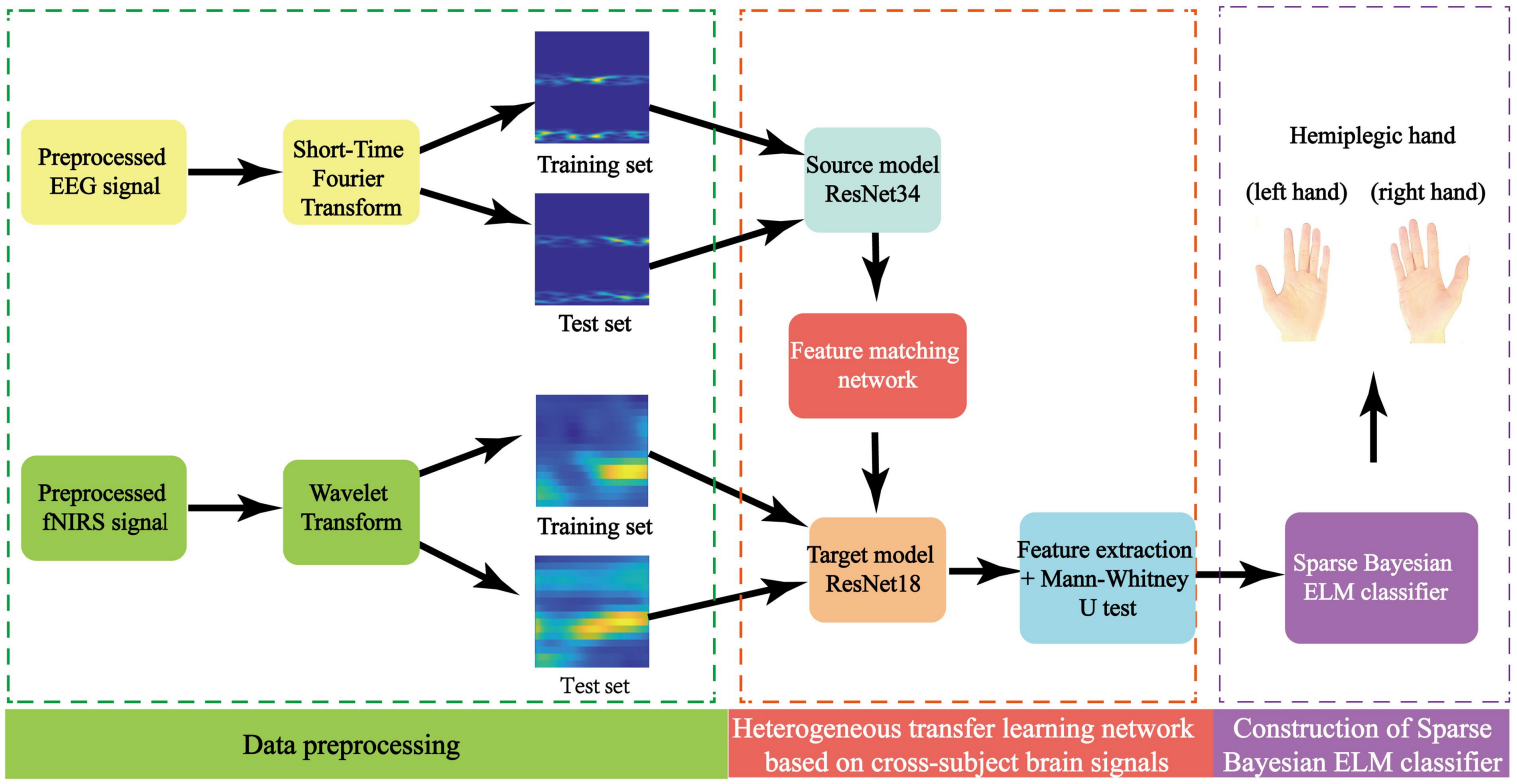
The algorithmic framework diagram of CHTLM.

The collection and annotation of functional near-infrared spectroscopy (fNIRS) data from stroke patients present substantial challenges, often leading to a limited number of training samples per subject. This scarcity of data restricts deep learning models from effectively capturing critical features necessary for accurate classification (Thippa Reddy et al., 2020). Additionally, significant physiological differences between patients further hinder the model’s ability to generalize across subjects, ultimately reducing predictive accuracy (Liu et al., 2023). To address these challenges, this study introduces a novel classification framework specifically designed for fNIRS-based motor imagery tasks—cross-subject motor imagery fNIRS signal Classification Algorithm Based on a Heterogeneous Transfer Learning Model (CHTLM). The overall structure of CHTLM is depicted in Figure 3. This framework overcomes the constraints of traditional classification methods that both small sample sizes and inter-subject variability.

CHTLM employs heterogeneous transfer learning, which allows knowledge transfer from a well-trained source domain model to strategically chosen layers of a target model. This adaptation enables the model to better generalize across patients and enhance classification accuracy. Following this, a multi-scale feature extraction mechanism processes fNIRS signals from stroke patients to capture both low- and high-level discriminative features. These extracted features are then passed through a Sparse Bayesian Extreme Learning Machine (BELM), which provides an efficient and robust classification solution, particularly suited for limited data scenarios. The CHTLM framework consists of three main components: (1) Feature matching network with adaptive selection—aligns source and target domain features dynamically to ensure effective knowledge transfer. (2) Feature extraction via ResNet—Captures spatial and temporal characteristics of fNIRS signals through hierarchical feature learning. (3) Sparse Bayesian Extreme Learning Machine (BELM) classifier—enhances classification robustness while reducing model complexity and computational cost. By integrating these components, CHTLM significantly improves the generalization and accuracy of motor imagery classification in stroke patients.

### Feature matching network based on adaptive selection

**Figure 4:**
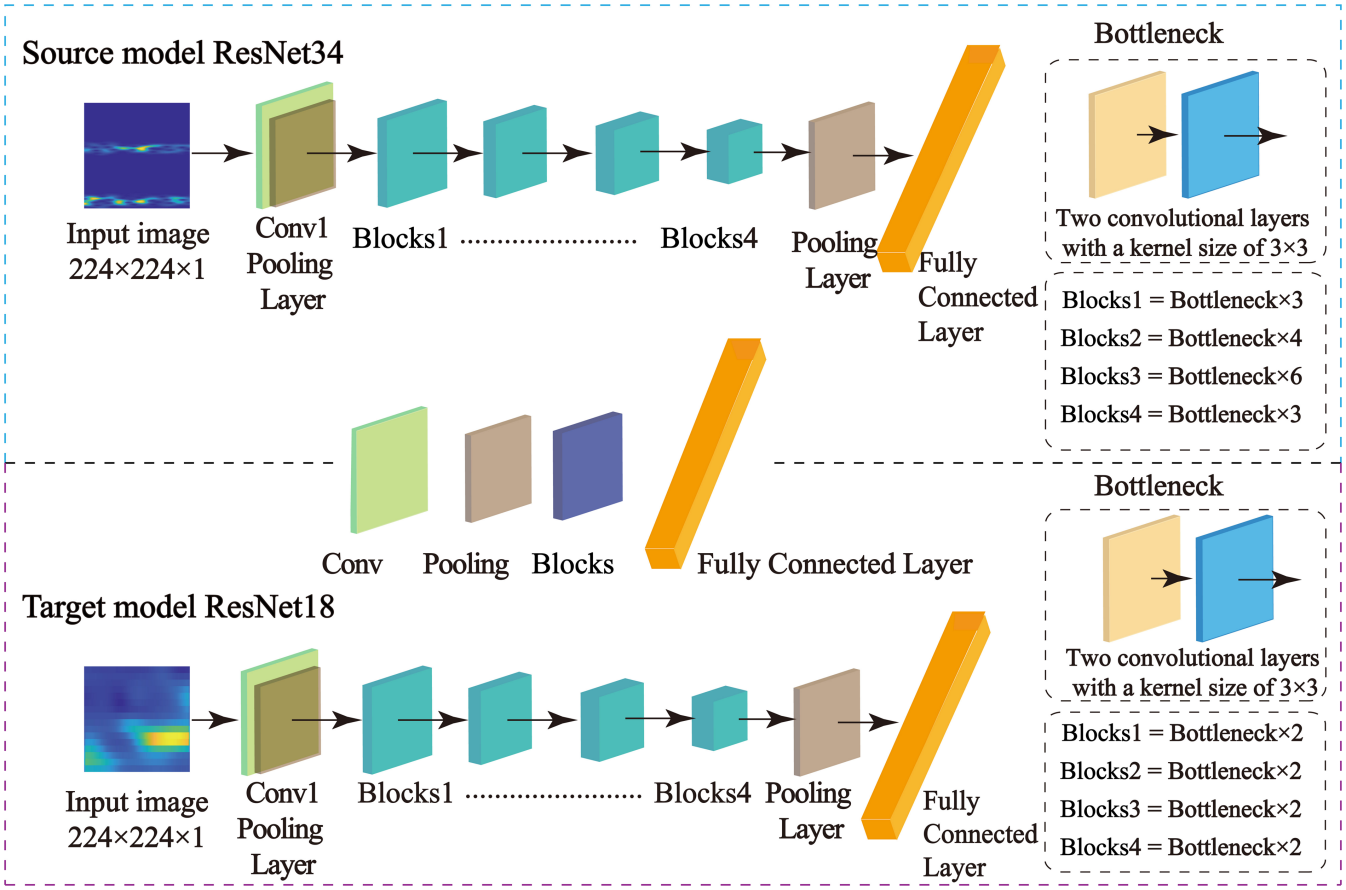
Structural diagrams of the source model and the target model.

In this study, the BCI Competition IV Dataset 2a served as the training data for the source domain model, while self-collected fNIRS data from stroke patients were used for the target model. Given the larger sample size of the source domain, a deeper architecture—ResNet34—was employed to fully exploit the dataset’s potential and enhance feature extraction. Conversely, the target model utilized a lighter ResNet18 architecture to reduce computational complexity and improve training efficiency. The overall model structure is depicted in Figure 4. Both models aim to extract rich, high-level feature representations from EEG and fNIRS signals, effectively learning deep features that are closely related to motor imagery. This approach facilitates the development of a cross-subject classification model with high accuracy. Details regarding the training parameter settings are provided in Supplementary material S4.

To enhance classification performance and improve generalizability, this study introduces a feature matching network (FMN) that dynamically selects and transfers relevant features from a pre-trained source model to strategically chosen layers in the target model. This adaptive feature-layer alignment ensures that only task-relevant information is transferred, preventing the introduction of redundant or misleading features that could degrade performance. In this study, the source model is trained on motor imagery (MI) tasks involving left- and right-hand movements in healthy individuals, while the target model is designed to classify MI tasks involving the paralyzed hand of stroke patients. The FMN first extracts multi-scale feature representations from the source model and then evaluates their relevance to the target task. It selectively transfers the most informative feature sets to corresponding layers in the target model, facilitating better adaptation to stroke patients’ MI signals. This process improves classification accuracy, particularly for distinguishing motor imagery involving the paralyzed hand.

**Figure 5:**
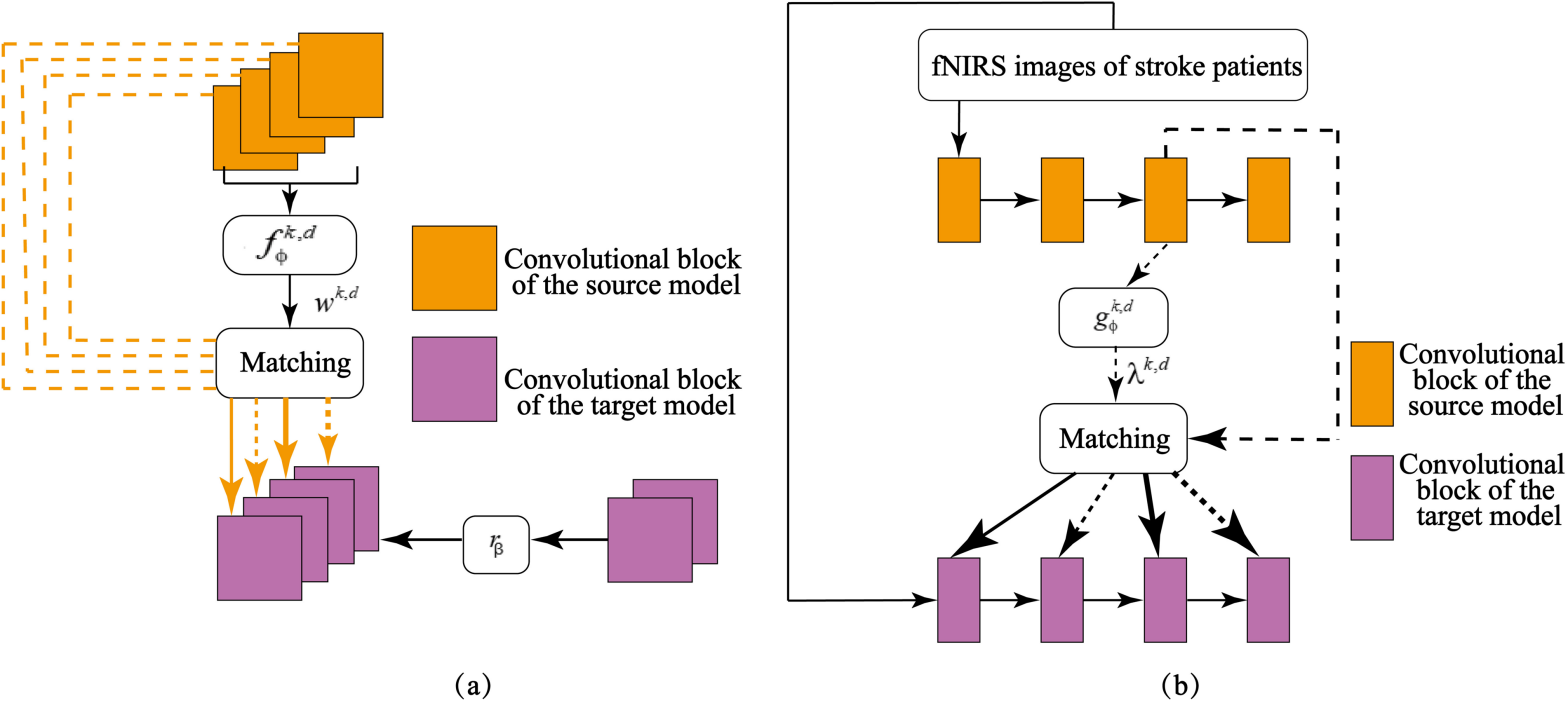
Adaptive feature matching network: **(a)** Selecting source knowledge useful for the target task; **(b)** Matching of feature layers between the target and source models.

The architecture of the feature matching network is illustrated in Figure 5, and it consists of two primary components, adaptive feature selection and layer-wise feature transfer. Adaptive feature selection (Figure 5a) identifies and extracts relevant feature representations from the source model that are beneficial for the target task. Layer-wise feature transfer (Figure 5b) integrates the selected features into optimally positioned layers of the target model, ensuring efficient adaptation to stroke patients’ MI signals. By effectively leveraging knowledge learned from healthy individuals, this transfer learning strategy enhances the target model’s ability to recognize MI signals in stroke patients, leading to improved cross-subject motor imagery classification accuracy.

The primary challenge in designing the feature-matching network is identifying which knowledge should be transferred from the source model to the target model. In heterogeneous transfer learning for cross-subject brain signal analysis, not all intermediate features from the source model contribute meaningfully to classifying motor imagery (MI) tasks involving the hemiplegic hand in stroke patients. To selectively transfer only the most relevant feature representations, this study introduces a weighted feature-matching loss mechanism. This approach quantifies the actual utility of each source feature map in the target task and assigns greater importance to features that enhance classification accuracy. Because different feature maps contribute unequally to the target model, a fully connected neural network is introduced to learn optimal weight values for each feature map. By processing the source model’s feature maps, this network automatically assigns importance scores, ensuring that the most informative features receive higher attention in the loss calculation. A fully connected neural network $f_{\phi }^{k,d}$ to learn the weights $w_{\theta }^{k,d}$ for each feature map in the target task. By inputting the feature maps from the source model into $f_{\phi }^{k,d}$, the weight values are obtained. As shown in Figure 5a, for a given set of feature maps from both the source model and the target model, trainable weights are assigned to each source feature map. Higher importance corresponds to greater weight values, allowing the model to prioritize essential features during transfer learning. This targeted selection of useful features enhances the effectiveness of knowledge transfer, ensuring that the model adapts well to MI signal classification in stroke patients.

Beyond selecting relevant features, the feature-matching network must determine where to transfer these features within the target model. Establishing layer-wise correlations between the source and target models is essential for effective knowledge transfer. By aligning feature representations from corresponding layers, the system ensures that critical MI knowledge learned from healthy individuals is mapped to the appropriate layers in the target model, thereby improving classification performance for hemiplegic hands in stroke patients. As depicted in Figure 5b, the adaptive feature-matching network facilitates this process by systematically mapping source-domain features to corresponding target-domain layers. Additional implementation details can be found in Supplementary material S5.

### Feature extraction based on convolutional neural networks

To effectively explore and utilize the multi-scale feature information in fNIRS and thus enhance the model’s accuracy. In the study, the convolutional kernels of the convolutional layers in the target model were used as feature extractors. Each feature extractor’s feature map was averaged to obtain the corresponding deep learning features from the fNIRS data. Through structural analysis of ResNet18 (He et al., 2016; Liu et al., 2023), we calculated that the network contains a total of 3,904 convolutional kernels. Therefore, 3,904 deep learning features were extracted from the fNIRS data for each subject. Furthermore, this study employed the Mann–Whitney *U* test (MacFarland and Yates, 2016) method to filter the fNIRS features, aiming to identify those that are highly relevant to the task and statistically significant. Details of feature extraction of the target model can be found in Supplementary material S6.

### Construction of Sparse Bayesian Extreme Learning Machine

**Figure 6:**
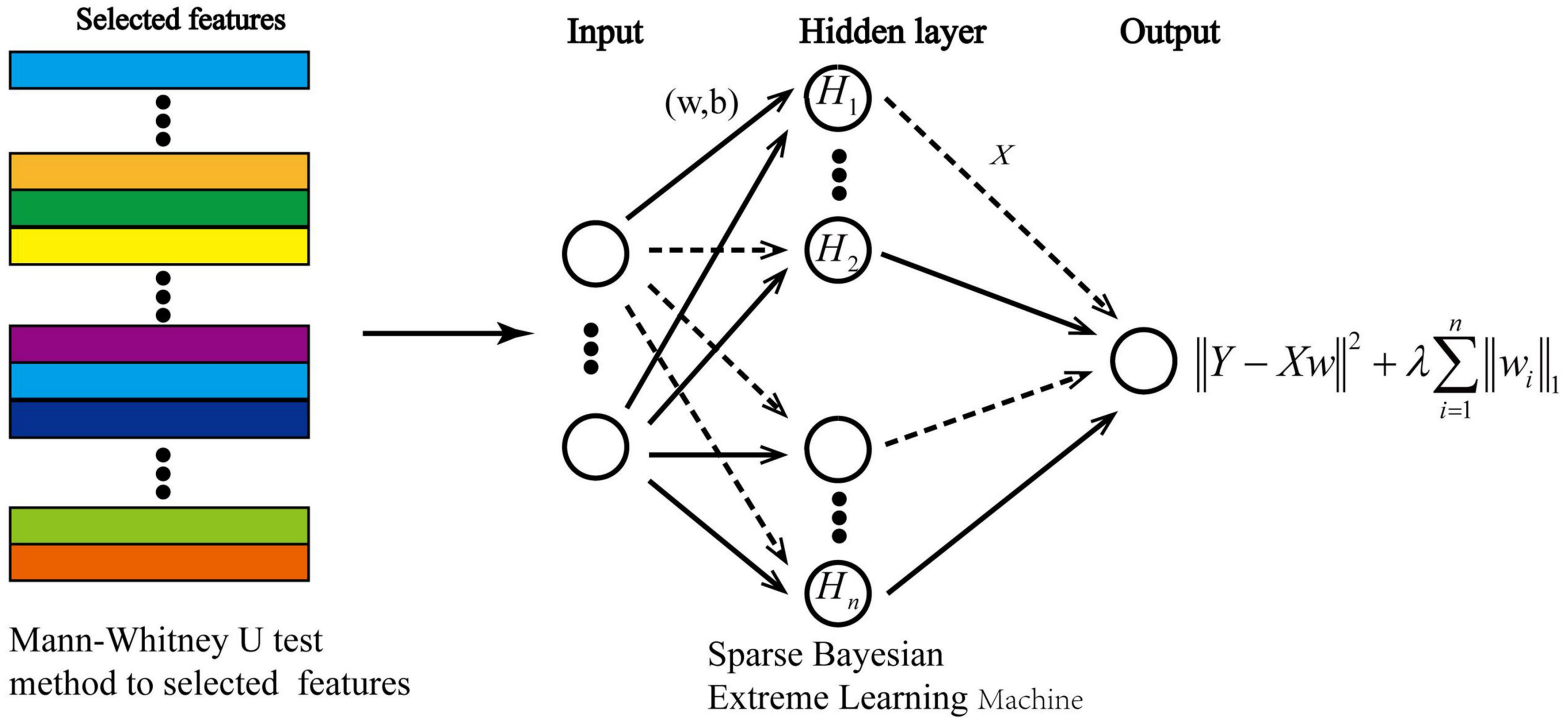
Construction of the classifier.

Bayesian Extreme Learning Machine (BELM) (Chaturvedi et al., 2018) is an algorithm that integrates Bayesian learning theory with Extreme Learning Machine (ELM) (Chouikh et al., 2021). This method utilizes the prior probability distribution inherent in Bayesian inference to randomly initialize the connection weights and biases between the input and hidden layers, resulting in a set of randomly parameterized neural networks. Subsequently, Bayesian inference is employed to estimate the posterior probability distribution of these parameters, allowing for fine-tuning of the weights and biases to achieve an optimal model solution. Compared to traditional approaches that manually set weights and biases, BELM offers a faster learning process, mitigates the overfitting issues associated with conventional ELM, and enhances the model’s generalization ability. To further improve the optimization and solution robustness of BELM, this study introduces an L1 norm constraint, promoting sparsity in the learned model parameters. Based on this approach, we construct a Sparse Bayesian Extreme Learning Machine (SBELM) classification model, as illustrated in Figure 6. Further details on the SBELM algorithm can be found in Supplementary material S7.

The Bayesian Extreme Learning Machine (BELM) (Chaturvedi et al., 2018) is an advanced neural network model that integrates Bayesian learning theory with Extreme Learning Machine (ELM) (Chouikh et al., 2021). Unlike conventional ELM, which manually initializes network weights and biases, BELM leverages Bayesian inference to randomly initialize these parameters while maintaining a prior probability distribution. Subsequently, posterior probability estimation is performed to refine the weights and biases, leading to an optimized classification model. Compared to standard ELM approaches, BELM offers: (1) Faster training speeds due to efficient parameter initialization. (2) Reduced overfitting, as Bayesian inference provides regularization. (3) Improved generalization, making it more robust for cross-subject MI classification tasks. To further enhance model sparsity and improve robustness, this study introduces an L1-norm constraint, promoting the selection of only the most relevant model parameters. This enhancement results in the construction of a Sparse Bayesian Extreme Learning Machine (SBELM) classification model, as illustrated in Figure 6. Further implementation details of SBELM can be found in Supplementary material S7. Furthermore, this study employed the Mann–Whitney *U* test method to filter the fNIRS features, aiming to identify those that are highly relevant to the task and statistically significant.

### Evaluation and comparison of models

To evaluate the model performance, the study used accuracy, area under the receiver operating characteristic curve (AUC), recall, and *F*_1_-score as performance metrics for the classification model. Among them, accuracy is used to evaluate the overall performance of the model on all categories. The motor imagery of the paralyzed hand is considered a positive case, while the resting state is a negative case. AUC is used to evaluate the model’s ability to correctly classify positive and negative cases. Recall assesses the model’s ability to identify positive cases. *F*_1_-score helps analyze the model’s balance in predicting positive and negative cases.

To validate the effectiveness of each module in CHTLM, five comparative experiments were conducted in this study: (1) Different transfer sources. The transfer source adopted by CHTLM is the motor imagery EEG data from the BCI Competition IV Dataset 2a, while the ImageNet-based Cross-Subject Heterogeneous Transfer Learning Model (I-CHTLM) uses the large-scale image dataset ImageNet (Deng et al., 2009) as the transfer source. (2) Different features. The features used by CHTLM are deep learning features extracted based on convolutional neural networks. The second set of experiments adopts six statistical features, forming a statistical feature-based heterogeneous transfer learning model (SF-HTLM). (3) Different classifiers. CHTLM employs SBELM as the classifier, while the third set of experiments uses ELM as the classifier, forming an ELM-based heterogeneous transfer learning model (E-HTLM). To evaluate the potential effectiveness of CHTLM, we conducted comparative experiments on datasets collected before and after rehabilitation training. (4) Different transfer learning models. CHTLM employs a heterogeneous transfer learning model and we compared it with two transfer learning models. The fourth set of experiments used an instance-based transfer learning model (ACTL), and the fifth set of experiments used a parameter-based transfer learning algorithm (TLCMI).

## Experiments and results

To evaluate the effectiveness of CHTLM, datasets from eight subjects (S1, S2, …, S8) were collected both before and after rehabilitation training. A leave-one-out cross-validation (LOO-CV) strategy (Cao et al., 2024) was employed in each experimental run, using fNIRS data from seven subjects as the training set and the remaining subject’s data as the test set. The detailed AUC and accuracy (ACC) results for different comparative models are provided in Supplementary material S8.

**Figure 7:**
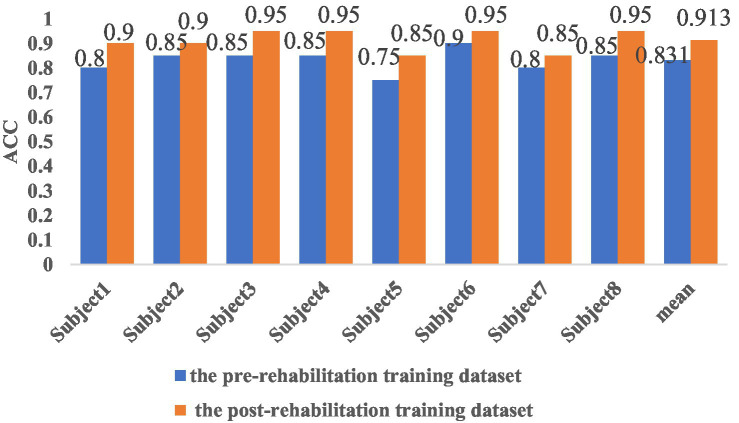
Comparison of classification accuracy before and after rehabilitation training for eight subjects.

The results demonstrate that CHTLM achieves high classification accuracy across both pre- and post-rehabilitation datasets. As shown in Figure 7, the average classification accuracy before rehabilitation was 0.831, increasing to 0.913 post-rehabilitation. Furthermore, subject-wise analysis indicated an improvement in classification accuracy ranging from 5 to 15%, suggesting a consistent enhancement in distinguishing motor imagery tasks after rehabilitation.

**Figure 8:**
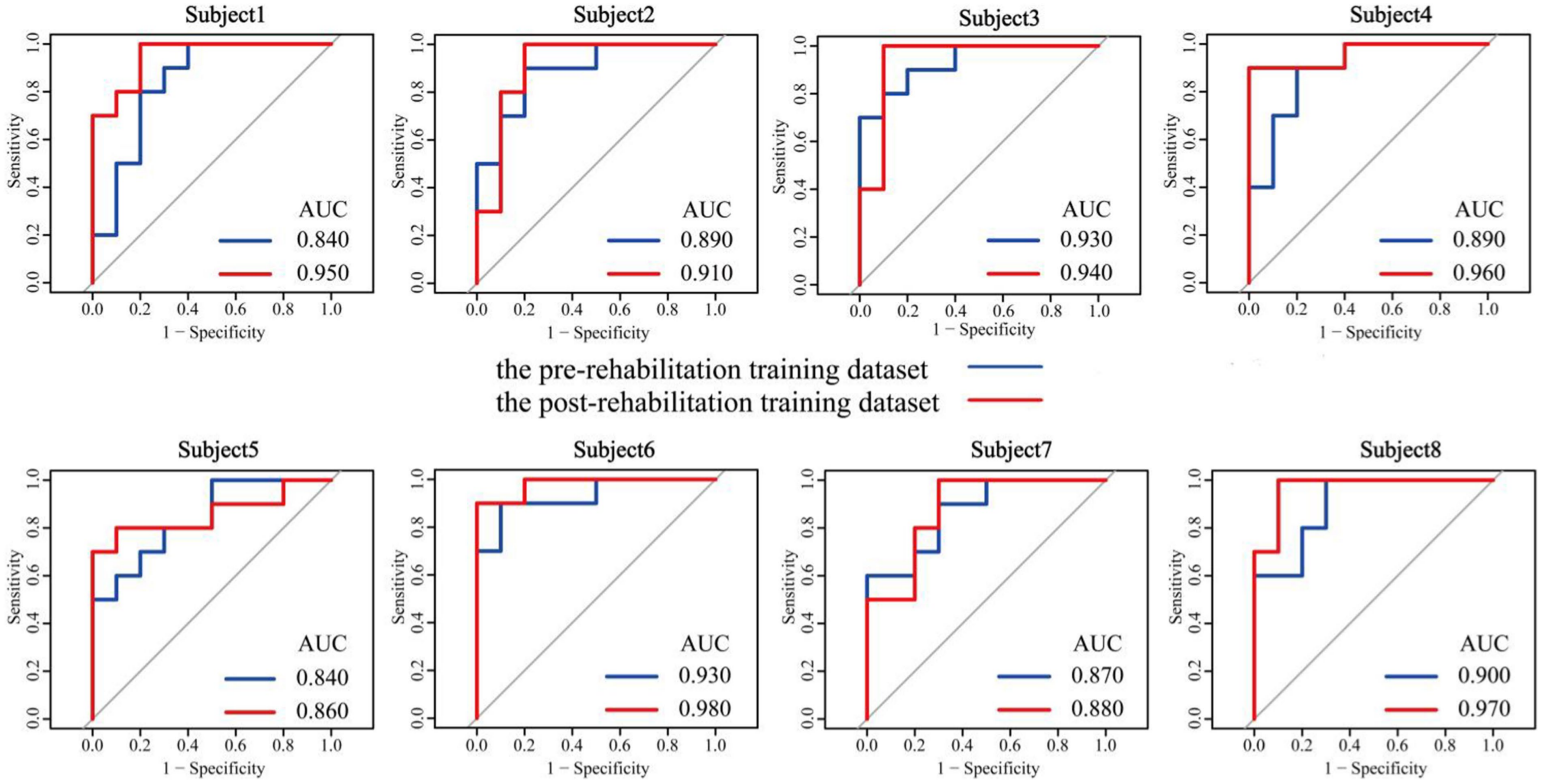
Comparison of AUC before and after rehabilitation training for eight subjects.

Figure 8 illustrates the receiver operating characteristic (ROC) curves of CHTLM for the eight subjects across both datasets. The mean AUC improved from 0.886 before rehabilitation to 0.931 post-rehabilitation. Additionally, individual subject AUC scores exhibited increases ranging from 1 to 11%. This improvement suggests that rehabilitation training contributes to varying degrees of brain function recovery, enabling the model to better differentiate motor imagery tasks for the left and right hands.

While statistical significance was not explicitly tested for these differences, the consistent improvement across all subjects supports the robustness of the model. Future studies could apply hypothesis testing, such as paired *t*-tests or chi-square test, to quantify the statistical significance of these improvements.

**Figure 9:**
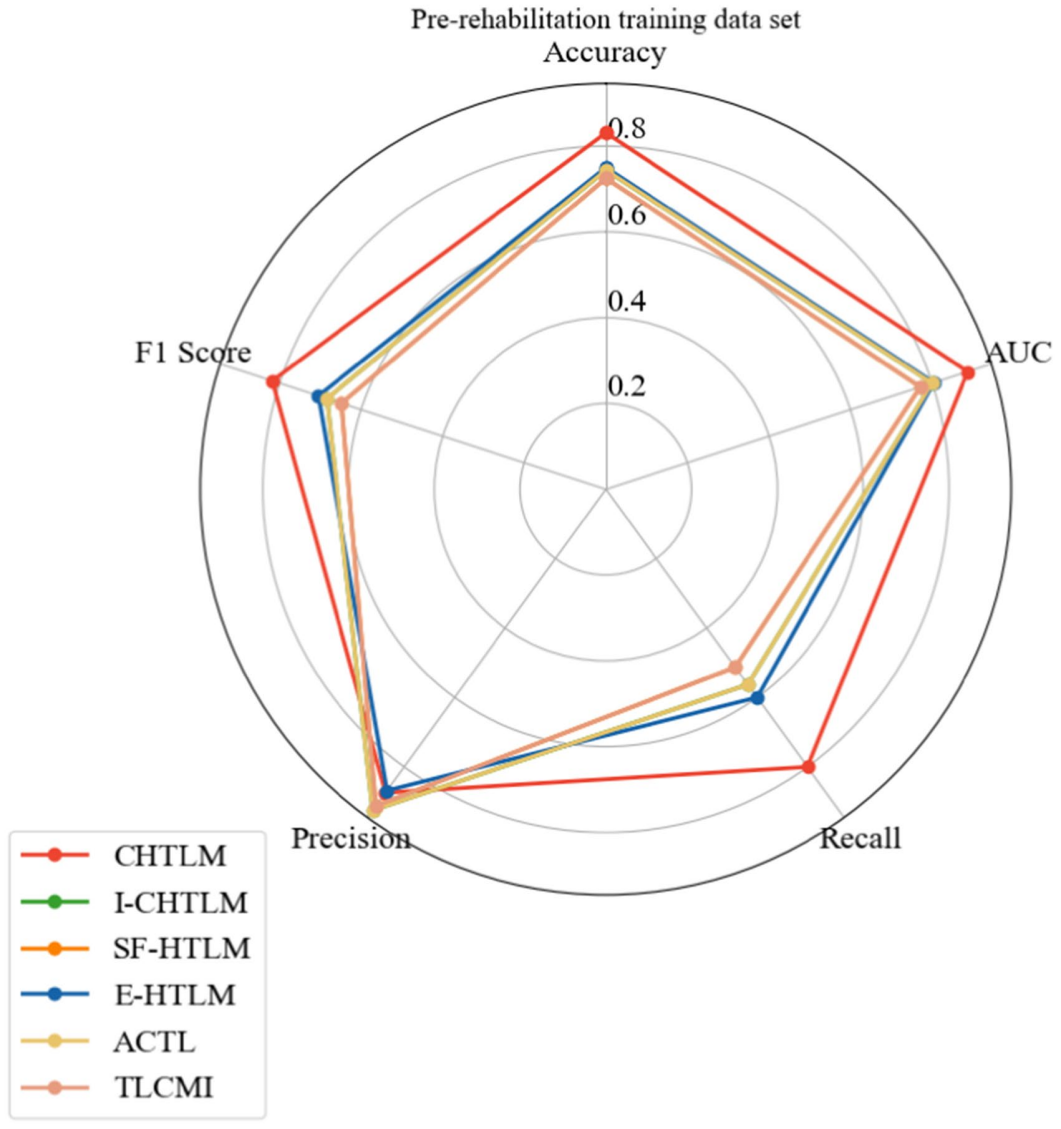
Radar plot of the pre-rehabilitation training dataset.

The mean performance metrics for the pre-rehabilitation dataset across the eight subjects are visualized in Figure 9 using a radar plot. This plot provides an intuitive comparison across five key indicators: accuracy, precision, recall, *F*_1_-score, and AUC value. Specific numerical values are detailed in Table 1. The radar chart shows that CHTLM exhibits the most balanced and robust performance, particularly excelling in accuracy (0.831), recall (0.800), and AUC (0.887). These metrics suggest that CHTLM not only maintains high classification accuracy but also achieves superior sensitivity in identifying motor imagery signals.

**Table 1:** Analysis of performance metrics for the comparative experiments on the pre-rehabilitation training dataset.

Subject	Method	Accuracy	AUC	Recall	Precision	*F*_1_-score
Mean	CHTLM	0.831	0.887	0.800	0.874	0.818
	I-CHTLM	0.744	0.800	0.563	0.925	0.684
	SF-HTLM	0.725	0.771	0.513	0.912	0.648
	E-HTLM	0.75	0.804	0.600	0.868	0.705
	ACTL	0.744	0.800	0.563	0.925	0.684
	TLCMI	0.725	0.771	0.513	0.912	0.648

Among the comparative models, E-HTLM demonstrated a relatively balanced performance, though its recall rate was notably lower, which may indicate a tendency towards false negatives. In contrast, I-CHTLM and ACTL models achieved high accuracy (0.925) but had smaller radar chart areas, suggesting weaker performance in other metrics such as recall and *F*_1_-score. The SF-HTLM and TLCMI models exhibited the smallest overall radar areas and the most imbalanced metric distributions, indicating inferior performance compared to CHTLM.

**Figure 10:**
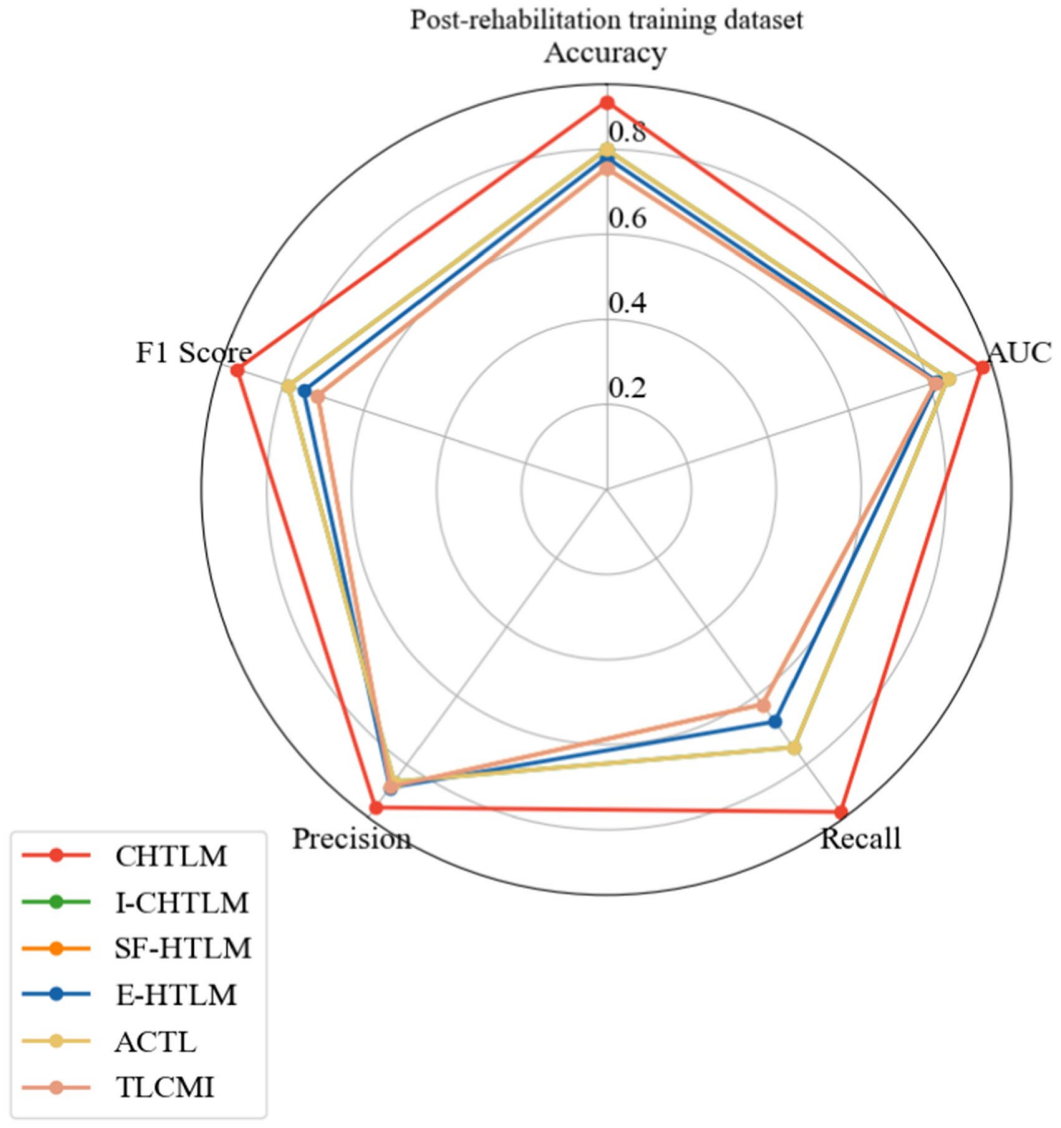
Radar plot of the post-rehabilitation training data set.

A similar radar analysis was conducted for the post-rehabilitation dataset (Figure 10), confirming the superior and well-rounded performance of CHTLM. Compared to the five baseline models, CHTLM consistently delivered the highest recall (0.938) and AUC (0.930), indicating its strong ability to capture relevant motor imagery patterns post-rehabilitation. The E-HTLM model performed well in terms of precision (0.867) and AUC (0.820), but its lower recall (0.675) and *F*_1_-score (0.750) suggest an imbalance between sensitivity and specificity. Likewise, I-CHTLM and ACTL exhibited strong precision (0.850) and AUC (0.846) but suffered from reduced recall. The SF-HTLM and TLCMI models, despite maintaining high accuracy (0.865), exhibited lower recall (0.625) and consequently a lower *F*_1_-score (0.715), limiting their overall effectiveness. To further substantiate these findings, future work should include statistical comparisons, such as confidence intervals or hypothesis testing on accuracy improvements, to validate the significance of the observed differences. Details of the specific performance indicators for each subject in the comparative models are provided in Supplementary material S9 (see Table 2).

**Table 2:** Analysis of performance metrics for the comparative experiments on the post-rehabilitation training dataset.

Subject	Method	Accuracy	AUC	Recall	Precision	*F*_1_-score
Mean	CHTLM	0.913	0.930	0.938	0.925	0.915
	I-CHTLM	0.800	0.846	0.750	0.850	0.788
	SF-HTLM	0.756	0.816	0.625	0.865	0.715
	E-HTLM	0.781	0.820	0.675	0.867	0.750
	ACTL	0.800	0.846	0.750	0.850	0.788
	TLCMI	0.756	0.816	0.625	0.865	0.715

## Discussion

Stroke is a prevalent neurological disorder that often results in hemiplegia, significantly impairing patients’ motor functions and quality of life (Gu and Huang, 2023). In hand rehabilitation therapy for stroke patients, traditional rehabilitation methods, such as passive training assisted by rehabilitation physicians, offer some benefits but are time-consuming, costly, and inherently limited (Baniqued et al., 2021; Shen et al., 2022). In contrast, the novel active rehabilitation approach based on motor imagery brain-computer interfaces (MI-BCI) has shown promising potential due to its ability to promote neural function recovery and neuroplasticity. Recent research on MI-BCI using functional near-infrared spectroscopy (fNIRS) has provided clinicians with a new tool to analyze the brain activity of stroke patients (Shen et al., 2022). Accurate classification of MI-fNIRS signals in stroke patients could yield deeper insights into their neural activity, aiding physicians in developing more precise and personalized rehabilitation treatment plans (Jiang et al., 2022). Moreover, improved classification methods may facilitate the development of more effective rehabilitation strategies, ultimately enhancing patients’ recovery processes.

In recent years, artificial intelligence (AI) has played a crucial role in the classification of MI brain signals. For instance, Dose et al. (2018) investigated MI EEG signals from 109 healthy individuals, utilizing one-dimensional convolutional neural network (CNN) layers to learn temporal and spatial filters for feature extraction. They then integrated these filters with traditional fully connected layers for classification, successfully developing an end-to-end MI EEG classification model. Similarly, Ma et al. (2021) explored various CNN-based time-series classification methods, applying them to MI fNIRS signals from 36 healthy individuals. However, due to significant inter-subject variability, models trained on data from one individual often fail to generalize well to other subjects, resulting in poor cross-subject classification performance [33]. This limitation poses challenges for physicians in designing personalized treatment plans. Additionally, although publicly available MI EEG datasets, such as the BCI Competition IV Dataset 2a, provide valuable resources, inconsistencies in data types and experimental tasks hinder the development and generalization of cross-subject classification models for stroke patients.

To validate the effectiveness of the proposed CHTLM model in cross-subject MI-fNIRS classification for stroke patients, we conducted comparative experiments against other transfer learning models. The experimental results demonstrated the superior performance of CHTLM, confirming its effectiveness in this classification task. Specifically, on two independent datasets collected before and after rehabilitation training, CHTLM achieved an average accuracy of 0.831 and 0.913, respectively, with corresponding AUC values of 0.887 and 0.930. Accurate classification of MI-fNIRS signals in stroke patients provides essential data for optimizing rehabilitation programs and improving patient outcomes. Additionally, validation on datasets from different rehabilitation stages further demonstrated the model’s generalization capability across varying recovery phases and individual differences.

In our comparative study, we first examined the impact of the transfer source on the classification model by comparing CHTLM with I-CHTLM. The experimental results showed a decline in performance across all metrics for I-CHTLM compared to CHTLM, with accuracy decreasing by 12.3% and AUC dropping by 9%. This decline is attributed to the use of the ImageNet dataset as a transfer source, which consists of large-scale image data that fundamentally differ from EEG signals at the feature level. When the source and target tasks are semantically dissimilar, heterogeneous transfer learning struggles to extract meaningful knowledge, leading to suboptimal model performance. The ACTL model achieved relatively high accuracy and AUC values (0.850 and 0.846, respectively), but its recall rate was relatively low (0.750), resulting in a suboptimal *F*_1_-score (0.788). Since ACTL relies on instance-based transfer learning, its performance is limited when the similarity between source and target domain instances is weak. In contrast, CHTLM leverages MI EEG data from healthy individuals as the transfer source, enabling it to capture deep features of healthy brain activity. Through an adaptive feature matching network, CHTLM effectively identifies correlations between source and target domains, allowing for the seamless transfer of relevant feature knowledge to the target model. The SF-HTLM model demonstrated the lowest performance across all metrics, with accuracy, AUC, recall, and *F*_1_-score values of 0.756, 0.816, 0.625, and 0.715, respectively. A comparison between CHTLM and SF-HTLM highlighted the advantage of deep learning-based feature extraction over traditional statistical methods, as statistical features fail to capture the deeper-level information present in EEG data. Similarly, the TLCMI model also exhibited the lowest performance, likely due to the inflexibility of its parameter-based transfer learning approach, which limits its adaptability to variations in the target task. By contrast, CHTLM not only captures hierarchical features but also, through an adaptive feature matching network, extracts deeper features that are closely associated with MI tasks. This approach effectively mitigates the impact of individual variability, enabling the model to achieve more robust cross-domain classification performance.

Additionally, a comparison between CHTLM and E-HTLM demonstrated that CHTLM, which integrates an Extreme Learning Machine (ELM) classifier, fully exploits the advantages of Bayesian inference, effectively incorporating the probability distribution of the data to improve classification accuracy. The inclusion of the L1 norm further enhances the model by producing sparse solutions, reducing complexity, and improving overall performance.

The proposed CHTLM algorithm offers several key advantages: (1) It leverages data from healthy individuals across different datasets and tasks through an adaptively selected feature matching network, significantly enriching the training knowledge of the target model. (2) It introduces a weighted feature matching loss that emphasizes source feature maps based on their relevance to the target task. Additionally, learnable parameters are assigned to each pair of feature maps in the feature matching network to quantify the degree of knowledge transfer from the source model to the target model. (3) By utilizing CNNs, CHTLM extracts multi-scale deep learning features that are highly relevant to MI classification, enhancing model generalizability and mitigating the impact of individual differences. Beyond its technical advantages, CHTLM demonstrates strong classification accuracy and generalization ability, likely due to the positive role of rehabilitation training in enhancing patients’ brain signal clarity. After rehabilitation, patients’ feature spaces become more aligned with those of healthy individuals, facilitating improved knowledge transfer. Ultimately, CHTLM provides a novel approach to knowledge transfer between healthy individuals and stroke patients, offering new insights and tools for personalized diagnosis and treatment in stroke rehabilitation. Experimental results confirm that CHTLM significantly outperforms competing methods both before and after rehabilitation, underscoring its potential in tracking rehabilitation progress and assessing treatment efficacy.

Despite its advantages, this study has several limitations that should be acknowledged: (1) To ensure patient comfort and minimize fatigue during experiments, the number of trials collected was relatively small. Consequently, the dataset may not fully capture the neurological diversity of stroke patients, potentially introducing biases in the data collection process. In particular, the high inter-subject variability among stroke patients may lead to results that are not fully generalizable. Future studies should aim to increase the number of trials per participant and expand the dataset through multi-center collaborations. This will not only mitigate potential biases but also enhance the robustness and generalizability of the model. (2) The current study is based on offline data analysis, which, while valuable for initial validations, does not fully represent real-time clinical scenarios. The offline nature of the experiments may overlook challenges such as latency and dynamic environmental factors that could affect model performance in an online setting. Future research should incorporate real-time signal processing and adaptive calibration strategies—such as dynamic domain adaptation—to validate CHTLM’s effectiveness in practical, clinical applications. We plan to develop a prototype system in collaboration with hospitals and perform dynamic feedback experiments to assess online performance and optimize treatment strategies for stroke patients. (3) Besides the small sample size and offline nature of the current experiments, other limitations include the challenges in managing inter-subject variability and potential data bias. These issues can affect the accuracy and reliability of motor imagery classification in stroke patients. To overcome these challenges, future work will focus on: real-time signal processing, enhanced data collection protocols, algorithmic optimizations. By addressing these limitations and outlining clear strategies for future research, we aim to bridge the gap between current offline analyses and real-time clinical applications, ultimately enhancing the clinical impact of our findings.

## Conclusion

Collecting and annotating fNIRS data from stroke patients presents significant challenges, often resulting in an insufficient number of training samples per subject. This data scarcity negatively impacts the predictive performance of deep learning models. Additionally, considerable physiological differences between individuals lead to poor cross-subject generalization of trained models. To address these limitations, this study proposes the CHTLM algorithm, which integrates an adaptive feature matching network and a sparse Bayesian-based ELM classifier.

First, we train a source model based on EEG data and a target model based on fNIRS data. Leveraging the feature extraction capabilities of ResNet, the model aims to capture complex relationships and intrinsic patterns across different subjects. Next, an adaptive feature matching network is introduced to align the feature representations of the source and target models. By selectively transferring relevant knowledge from the source domain to the target model, this approach mitigates inter-subject variability and enhances the model’s ability to generalize across individuals. Beyond its technical contributions, CHTLM facilitates knowledge transfer between healthy individuals and stroke patients, offering new insights and tools for personalized stroke diagnosis and rehabilitation. Experimental results demonstrate that CHTLM significantly outperforms comparative methods both before and after rehabilitation, highlighting its potential for tracking rehabilitation progress and assessing treatment efficacy.

## Data Availability

The original contributions presented in the study are included in the article/Supplementary material, further inquiries can be directed to the corresponding authors.
