# Supplementary material for: Heterogeneous transfer learning model for improving the classification performance of fNIRS signals in motor imagery among cross-subject stroke patients
Source: Front Hum Neurosci. 2025 Mar 27;19:1555690. doi: 10.3389/fnhum.2025.1555690 (PMC11983500; doi:10.3389/fnhum.2025.1555690)
Supplement: Supplementary file 1 [file Data_Sheet_1.docx]

**Supplemental Materials**

**Supplementary S1: Source Domain Data Collection**

In Dataset 2a, EEG signals were collected from 9 subjects using 22 EEG electrodes, in addition to electrooculography (EOG) signals recorded from 3 EOG electrodes. The sampling rate was set to 250 Hz. The distribution of the EEG electrodes is shown in Figure [引言] (a), while the distribution of the EOG electrodes is depicted in Figure [引言] (b).


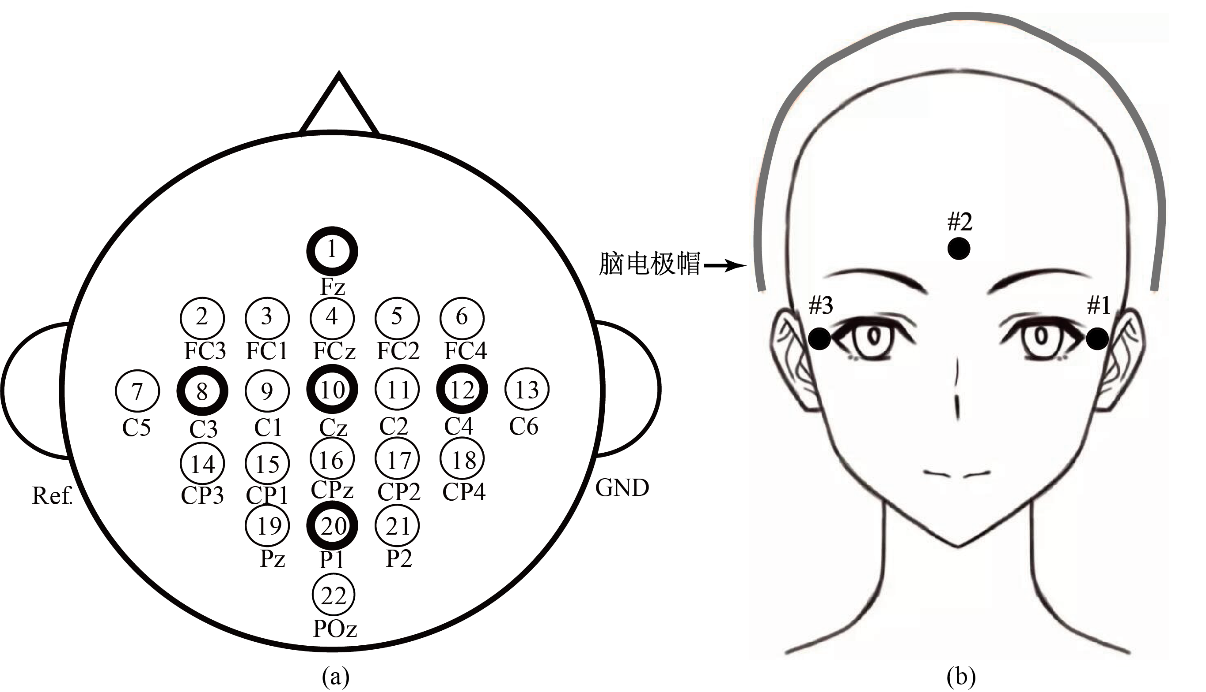


Figure 1 Distribution of electrode positions: (a) EEG; (b) Electrooculography

**Supplementary S2: Detailed Specifications of the Equipment**

In a publicly available EEG-fNIRS dataset, data were collected from 29 healthy subjects, comprising two independent datasets: (1) Dataset A (covering left-hand and right-hand MI tasks) and (2) Dataset B (including MA tasks and resting state data)[引言]. Both datasets contain EEG and fNIRS data. In this study, only EEG and fNIRS data related to left-hand and right-hand motor imagery tasks were used. In the publicly available MI-fNIRS dataset, signals were acquired using wavelengths of 760 nm and 850 nm at a sampling rate of 12.5 Hz. A total of 14 light sources and 16 detectors were arranged on the scalp, forming 36 channels, with a source-detector distance of 30 mm. In the publicly available MI-EEG dataset, data were collected using 30 active electrodes at a sampling rate of 1000 Hz.

**Supplementary S3: Preprocessing of MI-EEG Data**

The preprocessing steps for MI-EEG data in this study mainly included channel selection, band-pass filtering, and time window segmentation. The detailed steps are as follows:

- **Channel Selection**: This study focused on EEG data from the C3 and C4 channels to reduce the computational complexity of the source model while concentrating on information related to the motor cortex.
- **Band-pass Filtering**: During motor imagery, the relevant rhythmic signals include the $\mu$ rhythm (8-12 Hz) and $\beta$ rhythm (13-30 Hz). Therefore, an 8-30 Hz band-pass filter was applied to the selected EEG channels to filter out artifacts such as heartbeats and noise, thereby enhancing the signal-to-noise ratio.
- **Time Window Processing**: According to the experimental paradigm, each trial includes a 4-second motor imagery task. In this study, EEG data were extracted from the time window of 0.5-3.5 seconds after the cue, ensuring that the extracted data captured the main part of the motor imagery task while avoiding signal distortion caused by delay. Through this method, a 3-second time window of data was extracted for each trial, meeting the requirements for subsequent analysis and improving the overall efficiency of data processing.

Image-based Time-Frequency Representation of Brain Signals

To meet the input requirements of both the target and source models, this study used an image-based approach to represent fNIRS and EEG signals. Given the significant differences in data structure and characteristics between EEG and fNIRS, different signal processing methods were chosen for visualization.

**EEG Signal Representation**: EEG signals capture the activity of brain neurons, typically presented as time-domain signals that reflect changes over time. Due to the non-stationary nature of EEG signals, their high temporal resolution, and strong correlation with rhythmic variations, this study transformed EEG signals from the time domain to the time-frequency domain. Short-Time Fourier Transform (STFT), a widely used time-frequency analysis method, was selected, allowing for simultaneous analysis in both the time and frequency domains[引言]. STFT was applied to the EEG signals for image-based time-frequency representation due to its ability to accurately capture the signal variations in both time and frequency.

**fNIRS Signal Representation**: fNIRS signals are primarily used to measure changes in brain oxygen levels and typically include multiple channels, with each channel represented as a two-dimensional time series. The high spatial resolution of fNIRS allows clear depiction of variations across different brain regions. Given the flexibility of wavelet transforms in handling time series data, this study applied wavelet transforms for image-based time-frequency representation of fNIRS signals, enabling a clearer observation of time-frequency characteristics and meeting the input requirements of the target model[引言]. By applying one-dimensional wavelet transforms in both horizontal and vertical directions of the fNIRS data, it accurately captured changes across different channels and sampling points.

STFT-based EEG Images

In this study, EEG data were processed using Short-Time Fourier Transform (STFT) to extract time-frequency feature maps as the image representation of EEG signals. By utilizing the STFT algorithm, the EEG data were successfully converted into a time-frequency representation, forming a two-dimensional matrix with time and frequency axes. This transformation process facilitated the researchers’ ability to visually observe the temporal evolution characteristics of the signals within each time window, providing robust support for subsequent data analysis and network input. The specific procedure is described as follows[引言].

(1) Let the EEG data from the subjects be denoted as $x$. The input data for n subjects is represented as $X=\left(x_{1},x_{2},x_{3},\ldots\text{,}x_{n}\right)$.

(2)For any given subject, the data is segmented by time $\text{t}$, denoted as $x\left(t\right)$​

(3)The segmented data undergoes Short-Time Fourier Transform (STFT) to obtain the time-frequency feature map, represented as:

$x\left(\sigma\text{，}\beta\right)=\sum_{t} x\left(t\right)G\left(t-\beta\right)e^{j\sigma t}$ (1)

where:

$G\left(t-\beta\right)$​ is the window function (Hamming window used in this study).

$\beta$ and $\sigma$ denote time step and frequency band, respectively.

$j$ is the imaginary unit.

fNIRS Image Based on Wavelet Transform

In this study, discrete wavelet transform (DWT) was applied to fNIRS data to extract wavelet energy as the image representation of fNIRS signals. The fNIRS data consist of multi-channel time series in a two-dimensional format, where:

- The first dimension represents multiple channels, which are closely related.
- The second dimension represents the sampling points of each individual channel.

To obtain wavelet transform-based fNIRS images, this study applied a one-dimensional discrete wavelet transform algorithm along both the horizontal and vertical axes of the fNIRS data. Specifically:

1. **Horizontal Wavelet Transform**: A one-dimensional wavelet transform was first applied to the horizontal axis of the fNIRS data.
2. **Vertical Wavelet Transform**: The process was then repeated along the vertical axis.

By applying the transform across each row and column of the image, the fNIRS data were decomposed into three wavelet features representing intensity changes in the horizontal, vertical, and diagonal directions. These wavelet features correspond to the wavelet energy. The detailed procedure is as follow[引言]:

The local wavelet energies in three directions $E_{n}^{H}\left(x,y\right)$, $E_{n}^{V}\left(x,y\right)$, and $E_{n}^{D}\left(x,y\right)$ are defined as:

$\left\{\begin{matrix} E_{n}^{H}\left(x,y\right)=\sum_{k,b\in Z} \left(C_{H}\right)^{2}\frac{1}{2\pi\varepsilon}e^{-\left(\frac{\left(x-k\right)^{2}+\left(y-b\right)^{2}}{2\varepsilon^{2}}\right)} \\ E_{n}^{V}\left(x,y\right)=\sum_{k,b\in Z} \left(C_{V}\right)^{2}\frac{1}{2\pi\varepsilon}e^{-\left(\frac{\left(x-k\right)^{2}+\left(y-b\right)^{2}}{2\varepsilon^{2}}\right)} \\ E_{n}^{D}\left(x,y\right)=\sum_{k,b\in Z} \left(C_{D}\right)^{2}\frac{1}{2\pi\varepsilon}e^{-\left(\frac{\left(x-k\right)^{2}+\left(y-b\right)^{2}}{2\varepsilon^{2}}\right)} \end{matrix}\right.$ (2)

where:

- $Z$ is the set of integers.
- $n$ are the wavelet coefficients in the three directions.
- $C_{H}$、$C_{V}$ and $C_{D}$denotes the wavelet level.
- $\varepsilon$、$k$ and $b$ are the standard deviation, neighborhood sum, and coordinates, respectively.

**Logarithmic Average of Local Wavelet Energy Across n Wavelet Levels**:

$\left\{\begin{matrix} E_{H}\left(x,y\right)=\frac{1}{n}\sum_{1}^{n} \log E_{n}^{H}\left(x,y\right) \\ E_{V}\left(x,y\right)=\frac{1}{n}\sum_{1}^{n} \log E_{n}^{V}\left(x,y\right) \\ E_{D}\left(x,y\right)=\frac{1}{n}\sum_{1}^{n} \log E_{n}^{D}\left(x,y\right) \end{matrix}\right.$ (3)

**Total Wavelet Energy Across Three Directions**:

$E\left(x,y\right)=E_{H}\left(x,y\right)+E_{V}\left(x,y\right)+E_{D}\left(x,y\right)$ (4)

**Supplementary S4:**

### Data Collection and Experimental Setup

In this study, pre- and post-rehabilitation training datasets from 8 subjects (A1, A2, …, A8) were used. For each dataset, a leave-one-subject-out (LOSO) cross-validation method was employed, where fNIRS data from 7 subjects were used as the training set, and data from the remaining subject were used as the test set. The parameter settings for the cross-subject heterogeneous transfer learning model included three main parts:

**Source Model Based on EEG**:

- 1. Number of training epochs: 100
  2. Batch size per epoch: 64
  3. Momentum: 0.9
  4. Optimizer: Stochastic Gradient Descent (SGD)
  5. Loss function: Cross-entropy loss

**Target Model Based on fNIRS**:

- 1. Number of training epochs: 50
  2. Batch size per epoch: 64
  3. Learning rate: 0.1
  4. Feature matching term: set to 0.5
  5. Momentum: 0.9
  6. Optimizer: Adaptive learning rate optimization algorithm
  7. Loss function: Cross-entropy loss

The hardware used for this study was an NVIDIA GeForce RTX 2080 GPU with 24GB of memory, using CUDA version 12.1. The deep learning framework employed was PyTorch 1.8.1 GPU version, implemented in Python 3.6.

**Supplementary S5:**

Let$T_{d}(x)$and$S_{k}(x)$represent the feature maps of layer$d$in the target model and layer$k$in the source model, respectively, where$x$is the input matrix of the model. By calculating the$l_{2}$norm distance between the two, we can quantify the similarity or difference in features between the source model and the target model. This study aims to minimize this norm distance to promote the matching of features in the source model and the target model that are beneficial for learning the target task. Therefore, the overall learning objective of the feature matching network based on adaptive selection is:

$\left\|r_{\beta}(T_{d}^{\beta}(x))-S_{k}(x)\right\|_{2}^{2}$ (4)

Where$\beta$represents the hyperparameters of the target model, and$r_{\beta}$stands for the linear transformation.

Regarding the question of what knowledge to transfer, in the heterogeneous transfer network for cross-subject brain signal analysis, not all intermediate features from the source models are practically meaningful for the classification task of hemiplegic hands in stroke patients in the target model. To more precisely focus on those feature maps that are truly beneficial for the target task, this study introduces a weighted feature matching loss. By calculating the actual utility of each source feature map on the target task, it selectively emphasizes them, thus achieving an optimized selection of useful features. The weighted feature matching loss is defined as:

$L_{wfk}^{k,d}(\beta\left|x,w^{k,d}\right.)=\frac{1}{M\times N}\sum_{\theta} w_{\theta}^{k,d}\sum_{i,j} (r_{\beta}(T_{d}^{\beta}(x))_{\theta,i,j}-S_{k}(x)_{\theta,i,j})^{2}$ (5)

Where, $w_{\theta}^{k,d}$represents the non-negative weights for channel $\theta$, and $\sum_{\theta} w_{\theta}^{k,d}=1\text{，}w_{\theta}^{k,d}>0$. $M\times N$is the size of the feature maps from $S_{k}(x)$ and $r_{\beta}(T_{\text{d}}^{\beta}(x))$.

Since the importance of each feature map from the source model is not equal for the target task, this paper introduces a fully connected meta network to learn the weights$w_{\theta}^{k,d}$for each feature map in the target task. By inputting the feature maps from the source model into , the weight values are obtained. The relationship expression is:

(6)

Whererepresents the parameters of the network , and thus the weight values are determined by. For a given set of feature maps from the source model and the target model, different trainable weights need to be assigned to each feature map of the source model. The more significant the importance, the greater the corresponding weight value, thus allowing these feature maps to receive more attention in the loss calculation. By doing so, the model can more precisely focus on the features that are beneficial for the target task, enhancing the effectiveness of transfer learning.

Addressing the question of where to migrate the knowledge, we need to focus on the correlation between layers in the source model and the target model. This allows us to effectively transfer the key feature knowledge of motor imagery in healthy individuals learned from the source model to the target model, optimizing its performance in classifying motor imagery tasks for hemiplegic hands in stroke patients. We consider the output of each convolutional block as an independent unit and configure learnable parameters $\lambda^{\text{k},\text{d}}>0$ for each pair $\left(k\text{，}d\right)$of feature maps corresponding to the source and target models. These parameters quantify the degree of knowledge transfer from the source model's feature maps to the target model's feature maps. A higher value of this parameter indicates a greater potential for knowledge transfer between these feature map pairs. To learn these parameters, this paper introduces a fully connected meta network . The feature maps from the source model are input into to obtain the parameter values. The relational expression is:

(7)

The network possesses the ability of adaptive learning, enabling it to accurately identify the importance of feature maps from the source model relative to those of the target model. This ensures a more precise and efficient process of knowledge transfer. Consequently, a combined transmission loss can be obtained based on the combination of the weights $w_{\theta}^{k,d}$and the matching pair weights $\lambda^{\text{k},\text{d}}$:

(8)

Where the set $H$contains all pairs of convolutional blocks. Therefore, the final loss for the target model is

$L_{total}(\beta\left|x,y,f\right.) =\beta L_{wfk}(\beta\left|x,f\right.)+L_{org}(\beta\left|x,y\right.)$ (9)

Where $L_{org}(\beta\left|x,y\right.)$is the original loss from the target model ResNet18[17].

**Supplementary S6:**

illustrates the feature extraction process for fNIRS signals.


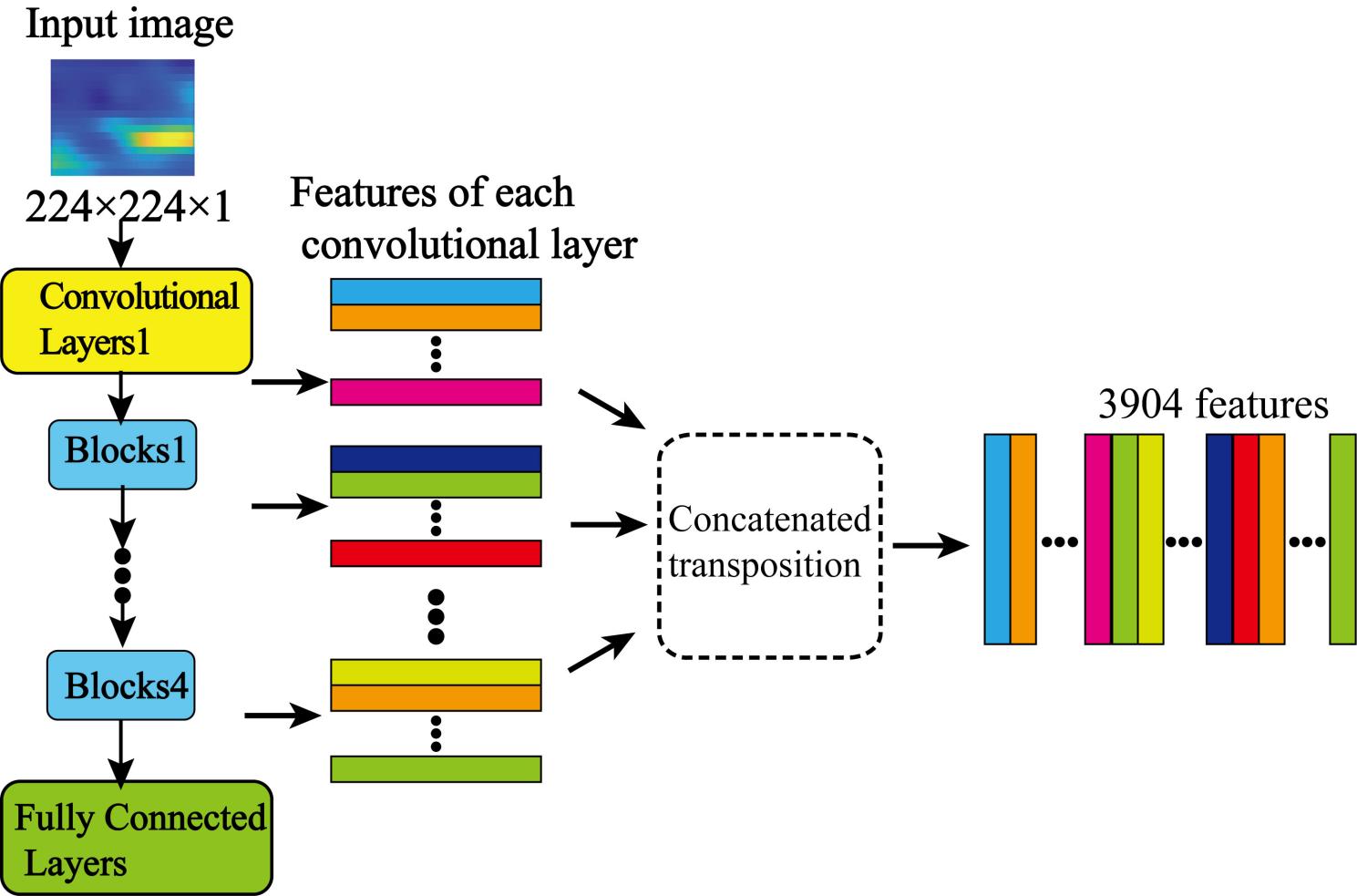


**Supplementary S7: SBELM**

The specific expression of the model is as follows:

$\mathrm{argmin}E(w,b)=\left\|Y-Xw\right\|^{2}+\lambda\sum_{i=1}^{n} \left\|w_{i}\right\|_{1}$ (10)

where:

- $Y$ is the predicted sample label,
- $X$ is the output of the hidden layer,
- $n$ is the number of neurons in the hidden layer,
- $w$ represents the weights between the hidden layer and the output layer,
- $\lambda$ is the regularization term coefficient, which controls the $l_{1}$ norm constraint.$\lambda>0$

### Likelihood Function

In solving this model, it is first necessary to assume a noise distribution. This study assumes the noise follows an inverse variance distribution. Let the inverse variance be denoted as $\theta$, where $y$ represents the result of Gaussian noise linearly added to the model output. Then, the likelihood function of the sample data $(X,t)$ with respect to the $w$ can be derived as follows:

$P(y|X,w,\theta)=N(y|x^{T}w,\theta^{-1})$ (11)

The sparse prior, posterior probabilities, covariance, and mean of the weights are given by:

$\left\{\begin{matrix} P(w|\alpha)=\prod_{K=1}^{n} N(w_{i}|0\text{，}\alpha_{i}^{-1}) \\ P(w|y,X,\alpha,\theta)=N(w|m,\varepsilon) \\ \varepsilon^{-1}=diag(\alpha)+\theta X^{T}X \\ m=\theta\varepsilon X^{T}y \end{matrix}\right.$ (12)

where:

- $\alpha=\left[\alpha_{1},\alpha_{2},...,\alpha_{n}\right]^{T}$ is the vector of hyperparameters,
- $diag$ is the diagonal matrix form.

$\alpha$、$\theta$can be updated using the marginal maximum likelihood estimation method.During the solution, marginal maximum likelihood estimation is used and specific iterative formulas are obtained.

$\left\{\begin{matrix} \alpha_{i}^{j+1}=\frac{1-\alpha_{i}^{j}\varepsilon_{ii}}{m_{i}^{T}m_{i}} \\ (\theta^{j+1})^{-1}=\frac{\left\|y-Xm\right\|^{2}}{N-\sum_{i=1}^{n} (1-\alpha_{k}^{j}\varepsilon_{KK})} \end{matrix}\right.$ (13)

Where $\varepsilon_{ii}$ is the i-nd diagonal component of the posterior distribution covariance $\varepsilon$, and $m_{i}$ is the i-th component of the posterior mean $m$.

In this study, to maximize the log-likelihood function, the following iterative training steps are required: first, set the initial values for parameters $\alpha$ and $\sigma^{2}$ to ensure the stability and convergence of the iteration process. Secondly, calculate the hyperparameters $m$ and $\varepsilon$. Finally, assess convergence, primarily focusing on the weights; if the iteration process does not converge, meaning the results do not meet the preset precision requirements, update hyperparameters $\alpha$ and $m$, and calculate the latest hyperparameters $m$ and $\varepsilon$. When the iteration process converges, the optimal mean $m$ and covariance $\varepsilon$ of the Gaussian distribution are obtained.

**Supplementary S8: Results**

**Contrast the model**

1.CHTLM(Heterogeneous migration+SBELM)

| Pre-rehabilitation | 1 | 2 | 3 | 4 | 5 | 6 | 7 | 8 | Mean |
| --- | --- | --- | --- | --- | --- | --- | --- | --- | --- |
| Train _ACC | 0.821 | 0.864 | 0.936 | 0.857 | 0.814 | 0.914 | 0.829 | 0.857 | 0.862 |
| Test _ACC | 0.800 | 0.850 | 0.850 | 0.850 | 0.750 | 0.900 | 0.800 | 0.850 | 0.831 |
| Train _AUC | 0.886 | 0.939 | 0.978 | 0.921 | 0.876 | 0.966 | 0.905 | 0.932 | 0.925 |
| Test _AUC | 0.840 | 0.890 | 0.930 | 0.890 | 0.840 | 0.930 | 0.870 | 0.900 | 0.886 |

| Post-rehabilitation | 1 | 2 | 3 | 4 | 5 | 6 | 7 | 8 | Mean |
| --- | --- | --- | --- | --- | --- | --- | --- | --- | --- |
| Train _ACC | 0.936 | 0.914 | 0.979 | 0.957 | 0.871 | 0.964 | 0.907 | 0.979 | 0.937 |
| Test _ACC | 0.900 | 0.900 | 0.950 | 0.950 | 0.850 | 0.950 | 0.850 | 0.950 | 0.913 |
| Train _AUC | 0.988 | 0.954 | 0.996 | 0.988 | 0.909 | 0.994 | 0.964 | 0.995 | 0.974 |
| Test _AUC | 0.950 | 0.910 | 0.940 | 0.960 | 0.860 | 0.980 | 0.880 | 0.970 | 0.931 |

2.E-HTLM（Heterogeneous migration+ELM）

| Pre-rehabilitation | 1 | 2 | 3 | 4 | 5 | 6 | 7 | 8 | Mean |
| --- | --- | --- | --- | --- | --- | --- | --- | --- | --- |
| Train _ACC | 0.743 | 0.814 | 0.793 | 0.843 | 0.764 | 0.829 | 0.736 | 0.786 | 0.789 |
| Test _ACC | 0.700 | 0.750 | 0.750 | 0.800 | 0.700 | 0.800 | 0.700 | 0.750 | 0.744 |
| Train _AUC | 0.794 | 0.869 | 0.860 | 0.899 | 0.804 | 0.857 | 0.768 | 0.840 | 0.836 |
| Test _AUC | 0.770 | 0.830 | 0.820 | 0.840 | 0.750 | 0.830 | 0.730 | 0.810 | 0.798 |

| Post-rehabilitation | 1 | 2 | 3 | 4 | 5 | 6 | 7 | 8 | Mean |
| --- | --- | --- | --- | --- | --- | --- | --- | --- | --- |
| Train _ACC | 0.771 | 0.814 | 0.779 | 0.836 | 0.793 | 0.864 | 0.807 | 0.886 | 0.819 |
| Test _ACC | 0.750 | 0.800 | 0.750 | 0.800 | 0.750 | 0.800 | 0.750 | 0.850 | 0.781 |
| Train _AUC | 0.804 | 0.851 | 0.838 | 0.870 | 0.841 | 0.922 | 0.873 | 0.910 | 0.864 |
| Test _AUC | 0.760 | 0.800 | 0.790 | 0.830 | 0.830 | 0.870 | 0.830 | 0.850 | 0.820 |

3、SF-HTLM（statistical test +SBELM）

| Pre-rehabilitation | 1 | 2 | 3 | 4 | 5 | 6 | 7 | 8 | Mean |
| --- | --- | --- | --- | --- | --- | --- | --- | --- | --- |
| Train _ACC | 0.743 | 0.779 | 0.757 | 0.779 | 0.757 | 0.793 | 0.743 | 0.793 | 0.768 |
| Test _ACC | 0.700 | 0.750 | 0.700 | 0.750 | 0.700 | 0.750 | 0.700 | 0.750 | 0.725 |
| Train _AUC | 0.761 | 0.816 | 0.804 | 0.809 | 0.791 | 0.825 | 0.784 | 0.843 | 0.804 |
| Test _AUC | 0.750 | 0.790 | 0.750 | 0.800 | 0.740 | 0.770 | 0.750 | 0.820 | 0.771 |

| Post-rehabilitation | 1 | 2 | 3 | 4 | 5 | 6 | 7 | 8 | Mean |
| --- | --- | --- | --- | --- | --- | --- | --- | --- | --- |
| Train _ACC | 0.814 | 0.807 | 0.750 | 0.814 | 0.743 | 0.793 | 0.786 | 0.829 | 0.792 |
| Test _ACC | 0.750 | 0.750 | 0.700 | 0.800 | 0.700 | 0.750 | 0.750 | 0.800 | 0.750 |
| Train _AUC | 0.825 | 0.849 | 0.808 | 0.888 | 0.813 | 0.840 | 0.850 | 0.897 | 0.846 |
| Test _AUC | 0.780 | 0.820 | 0.760 | 0.860 | 0.770 | 0.820 | 0.810 | 0.850 | 0.809 |

4、I-CHTLM（Heterogeneous migration+SBELM+imagenet）

| Pre-rehabilitation | 1 | 2 | 3 | 4 | 5 | 6 | 7 | 8 | Mean |
| --- | --- | --- | --- | --- | --- | --- | --- | --- | --- |
| Train _ACC | 0.729 | 0.793 | 0.857 | 0.843 | 0.750 | 0.836 | 0.757 | 0.736 | 0.788 |
| Test _ACC | 0.700 | 0.750 | 0.800 | 0.800 | 0.700 | 0.800 | 0.700 | 0.700 | 0.744 |
| Train _AUC | 0.769 | 0.824 | 0.934 | 0.908 | 0.755 | 0.906 | 0.811 | 0.798 | 0.838 |
| Test _AUC | 0.730 | 0.800 | 0.890 | 0.860 | 0.720 | 0.890 | 0.760 | 0.750 | 0.800 |

| Post-rehabilitation | 1 | 2 | 3 | 4 | 5 | 6 | 7 | 8 | Mean |
| --- | --- | --- | --- | --- | --- | --- | --- | --- | --- |
| Train _ACC | 0.800 | 0.836 | 0.900 | 0.929 | 0.793 | 0.893 | 0.786 | 0.843 | 0.848 |
| Test _ACC | 0.750 | 0.800 | 0.850 | 0.900 | 0.750 | 0.800 | 0.750 | 0.800 | 0.800 |
| Train _AUC | 0.826 | 0.872 | 0.950 | 0.967 | 0.834 | 0.924 | 0.833 | 0.915 | 0.890 |
| Test _AUC | 0.780 | 0.850 | 0.900 | 0.910 | 0.810 | 0.870 | 0.790 | 0.860 | 0.846 |

5.ACTL(instance-based transfer learning)

| Pre-rehabilitation | 1 | 2 | 3 | 4 | 5 | 6 | 7 | 9 | Mean |
| --- | --- | --- | --- | --- | --- | --- | --- | --- | --- |
| Train _ACC | 0.850 | 0.814 | 0.793 | 0.821 | 0.743 | 0.807 | 0.821 | 0.879 | 0.816 |
| Test _ACC | 0.750 | 0.750 | 0.750 | 0.800 | 0.700 | 0.750 | 0.750 | 0.800 | 0.756 |
| Train _AUC | 0.939 | 0.839 | 0.837 | 0.884 | 0.804 | 0.900 | 0.878 | 0.941 | 0.878 |
| Test _AUC | 0.750 | 0.750 | 0.700 | 0.840 | 0.730 | 0.740 | 0.810 | 0.700 | 0.753 |

| Post-rehabilitation | 1 | 2 | 3 | 4 | 5 | 6 | 7 | 9 | Mean |
| --- | --- | --- | --- | --- | --- | --- | --- | --- | --- |
| Train _ACC | 0.921 | 0.921 | 0.814 | 0.936 | 0.814 | 0.843 | 0.821 | 0.921 | 0.874 |
| Test _ACC | 0.850 | 0.850 | 0.800 | 0.900 | 0.800 | 0.800 | 0.800 | 0.850 | 0.831 |
| Train _AUC | 0.982 | 0.969 | 0.892 | 0.986 | 0.889 | 0.868 | 0.894 | 0.972 | 0.932 |
| Test _AUC | 0.760 | 0.740 | 0.820 | 0.890 | 0.820 | 0.800 | 0.850 | 0.780 | 0.808 |

6.TLCMI( parameter-based transfer learning algorithm )

| Pre-rehabilitation | 1 | 2 | 3 | 4 | 5 | 6 | 7 | 9 | Mean |
| --- | --- | --- | --- | --- | --- | --- | --- | --- | --- |
| Train _ACC | 0.757 | 0.900 | 0.836 | 0.879 | 0.821 | 0.850 | 0.771 | 0.800 | 0.827 |
| Test _ACC | 0.700 | 0.700 | 0.750 | 0.750 | 0.700 | 0.700 | 0.700 | 0.700 | 0.713 |
| Train _AUC | 0.845 | 0.932 | 0.896 | 0.929 | 0.906 | 0.909 | 0.850 | 0.867 | 0.892 |
| Test _AUC | 0.700 | 0.650 | 0.690 | 0.710 | 0.640 | 0.620 | 0.660 | 0.560 | 0.654 |

| Post-rehabilitation | 1 | 2 | 3 | 4 | 5 | 6 | 7 | 9 | Mean |
| --- | --- | --- | --- | --- | --- | --- | --- | --- | --- |
| Train _ACC | 0.929 | 0.943 | 0.829 | 0.857 | 0.921 | 0.986 | 0.821 | 0.829 | 0.889 |
| Test _ACC | 0.850 | 0.750 | 0.800 | 0.850 | 0.800 | 0.800 | 0.750 | 0.750 | 0.794 |
| Train _AUC | 0.971 | 0.978 | 0.901 | 0.903 | 0.966 | 0.996 | 0.874 | 0.882 | 0.934 |
| Test _AUC | 0.800 | 0.660 | 0.780 | 0.770 | 0.700 | 0.670 | 0.700 | 0.700 | 0.722 |

**Supplementary S9: Results**

**Contrast experiment**

Table 1 Analysis of performance metrics for the comparative experiments on the pre-rehabilitation training dataset

| Subject | Method | Accuracy | AUC | Recall | Precision | F1 Score |
| --- | --- | --- | --- | --- | --- | --- |
| A1 | **CHTLM** | 0.800 | 0.840 | 0.800 | 0.800 | 0.800 |
|  | I-CHTLM | 0.700 | 0.730 | 0.600 | 0.800 | 0.667 |
|  | SF-HTLM | 0.700 | 0.750 | 0.400 | 1.000 | 0.571 |
|  | E-HTLM | 0.700 | 0.770 | 0.600 | 0.750 | 0.667 |
|  | ACTL | 0.750 | 0.750 | 0.700 | 0.800 | 0.737 |
|  | TLCMI | 0.700 | 0.700 | 0.800 | 0.600 | 0.727 |
| A2 | **CHTLM** | 0.850 | 0.890 | 0.900 | 0.818 | 0.857 |
|  | I-CHTLM | 0.7500 | 0.800 | 0.500 | 1.000 | 0.667 |
|  | SF-HTLM | 0.750 | 0.790 | 0.600 | 0.857 | 0.706 |
|  | E-HTLM | 0.750 | 0.830 | 0.600 | 0.857 | 0.706 |
|  | ACTL | 0.750 | 0.750 | 0.600 | 0.900 | 0.706 |
|  | TLCMI | 0.700 | 0.650 | 0.600 | 0.800 | 0.667 |
| A3 | **CHTLM** | 0.850 | 0.930 | 0.800 | 0.889 | 0.842 |
|  | I-CHTLM | 0.800 | 0.890 | 0.600 | 1.000 | 0.750 |
|  | SF-HTLM | 0.700 | 0.750 | 0.500 | 0.833 | 0.625 |
|  | E-HTLM | 0.750 | 0.820 | 0.500 | 1.000 | 0.667 |
|  | ACTL | 0.750 | 0.720 | 0.600 | 0.900 | 0.706 |
|  | TLCMI | 0.750 | 0.690 | 0.600 | 0.900 | 0.706 |
| A4 | **CHTLM** | 0.850 | 0.890 | 0.900 | 0.818 | 0.857 |
|  | I-CHTLM | 0.800 | 0.860 | 0.700 | 0.900 | 0.778 |
|  | SF-HTLM | 0.750 | 0.800 | 0.500 | 1.000 | 0.667 |
|  | E-HTLM | 0.800 | 0.840 | 0.700 | 0.875 | 0.778 |
|  | ACTL | 0.800 | 0.840 | 0.800 | 0.800 | 0.800 |
|  | TLCMI | 0.750 | 0.710 | 0.700 | 0.800 | 0.737 |
| A5 | **CHTLM** | 0.750 | 0.840 | 0.500 | 1.000 | 0.667 |
|  | I-CHTLM | 0.700 | 0.720 | 0.500 | 0.900 | 0.625 |
|  | SF-HTLM | 0.700 | 0.740 | 0.600 | 0.750 | 0.667 |
|  | E-HTLM | 0.700 | 0.750 | 0.600 | 0.750 | 0.667 |
|  | ACTL | 0.700 | 0.730 | 0.400 | 1.000 | 0.571 |
|  | TLCMI | 0.600 | 0.450 | 0.200 | 1.000 | 0.337 |
| A6 | **CHTLM** | 0.900 | 0.930 | 0.900 | 0.900 | 0.900 |
|  | I-CHTLM | 0.800 | 0.890 | 0.600 | 1.000 | 0.750 |
|  | SF-HTLM | 0.750 | 0.770 | 0.600 | 0.857 | 0.706 |
|  | E-HTLM | 0.800 | 0.830 | 0.600 | 1.000 | 0.750 |
|  | ACTL | 0.750 | 0.740 | 0.600 | 0.900 | 0.706 |
|  | TLCMI | 0.700 | 0.620 | 0.800 | 0.600 | 0.727 |
| A7 | **CHTLM** | 0.800 | 0.870 | 0.600 | 1.000 | 0.750 |
|  | I-CHTLM | 0.700 | 0.760 | 0.400 | 1.000 | 0.571 |
|  | SF-HTLM | 0.700 | 0.750 | 0.400 | 1.000 | 0.571 |
|  | E-HTLM | 0.700 | 0.730 | 0.500 | 0.833 | 0.625 |
|  | ACTL | 0.750 | 0.810 | 0.500 | 1.000 | 0.667 |
|  | TLCMI | 0.700 | 0.660 | 0.800 | 0.600 | 0.727 |
| A8 | **CHTLM** | 0.850 | 0.900 | 1.000 | 0.769 | 0.870 |
|  | I-CHTLM | 0.700 | 0.750 | 0.600 | 0.800 | 0.667 |
|  | SF-HTLM | 0.750 | 0.820 | 0.500 | 1.000 | 0.667 |
|  | E-HTLM | 0.800 | 0.860 | 0.700 | 0.875 | 0.778 |
|  | ACTL | 0.800 | 0.700 | 0.800 | 0.800 | 0.800 |
|  | TLCMI | 0.700 | 0.560 | 0.900 | 0.500 | 0.750 |
| Mean | **CHTLM** | 0.831 | 0.887 | 0.800 | 0.874 | 0.818 |
|  | I-CHTLM | 0.744 | 0.800 | 0.563 | 0.925 | 0.684 |
|  | SF-HTLM | 0.725 | 0.771 | 0.513 | 0.912 | 0.648 |
|  | E-HTLM | 0.75 | 0.804 | 0.600 | 0.868 | 0.705 |
|  | ACTL | 0.744 | 0.800 | 0.563 | 0.925 | 0.684 |
|  | TLCMI | 0.725 | 0.771 | 0.513 | 0.912 | 0.648 |

Table 2 Analysis of performance metrics for the comparative experiments on the post-rehabilitation training dataset

| Subject | Method | Accuracy | AUC | Recall | Precision | F1 Score |
| --- | --- | --- | --- | --- | --- | --- |
| A1 | **CHTLM** | 0.900 | 0.950 | 1.000 | 0.833 | 0.909 |
|  | I-CHTLM | 0.750 | 0.780 | 0.700 | 0.800 | 0.737 |
|  | SF-HTLM | 0.800 | 0.840 | 0.600 | 1.000 | 0.750 |
|  | E-HTLM | 0.750 | 0.760 | 0.600 | 0.857 | 0.706 |
|  | ACTL | 0.850 | 0.760 | 0.700 | 1.000 | 0.824 |
|  | TLCMI | 0.850 | 0.800 | 1.000 | 0.700 | 0.870 |
| A2 | **CHTLM** | 0.900 | 0.910 | 1.000 | 1.000 | 0.909 |
|  | I-CHTLM | 0.800 | 0.850 | 0.700 | 0.900 | 0.778 |
|  | SF-HTLM | 0.750 | 0.820 | 0.500 | 1.000 | 0.667 |
|  | E-HTLM | 0.800 | 0.800 | 0.700 | 0.875 | 0.778 |
|  | ACTL | 0.850 | 0.740 | 1.000 | 0.700 | 0.870 |
|  | TLCMI | 0.750 | 0.660 | 0.700 | 0.800 | 0.737 |
| A3 | **CHTLM** | 0.950 | 0.930 | 0.900 | 1.000 | 0.947 |
|  | I-CHTLM | 0.850 | 0.900 | 0.800 | 0.900 | 0.842 |
|  | SF-HTLM | 0.700 | 0.760 | 0.500 | 0.833 | 0.625 |
|  | E-HTLM | 0.750 | 0.790 | 0.600 | 0.857 | 0.706 |
|  | ACTL | 0.800 | 0.820 | 0.900 | 0.700 | 0.818 |
|  | TLCMI | 0.800 | 0.780 | 0.800 | 0.800 | 0.800 |
| A4 | **CHTLM** | 0.950 | 0.960 | 0.900 | 1.000 | 0.947 |
|  | I-CHTLM | 0.900 | 0.910 | 0.800 | 1.000 | 0.899 |
|  | SF-HTLM | 0.800 | 0.860 | 0.700 | 0.875 | 0.778 |
|  | E-HTLM | 0.800 | 0.830 | 0.700 | 0.875 | 0.778 |
|  | ACTL | 0.850 | 0.870 | 0.900 | 0.800 | 0.857 |
|  | TLCMI | 0.850 | 0.770 | 0.800 | 0.900 | 0.842 |
| A5 | **CHTLM** | 0.850 | 0.860 | 0.800 | 0.889 | 0.842 |
|  | I-CHTLM | 0.750 | 0.810 | 0.500 | 1.000 | 0.667 |
|  | SF-HTLM | 0.700 | 0.770 | 0.600 | 0.750 | 0.667 |
|  | E-HTLM | 0.750 | 0.830 | 0.600 | 0.857 | 0.706 |
|  | ACTL | 0.800 | 0.820 | 0.700 | 0.900 | 0.778 |
|  | TLCMI | 0.800 | 0.700 | 0.800 | 0.800 | 0.800 |
| A6 | **CHTLM** | 0.950 | 0.980 | 0.900 | 1.000 | 0.947 |
|  | I-CHTLM | 0.800 | 0.870 | 0.700 | 0.900 | 0.778 |
|  | SF-HTLM | 0.750 | 0.820 | 0.600 | 0.857 | 0.706 |
|  | E-HTLM | 0.800 | 0.870 | 0.800 | 0.800 | 0.800 |
|  | ACTL | 0.800 | 0.800 | 0.900 | 0.700 | 0.818 |
|  | TLCMI | 0.800 | 0.670 | 0.800 | 0.800 | 0.800 |
| A7 | **CHTLM** | 0.850 | 0.880 | 1.000 | 0.769 | 0.870 |
|  | I-CHTLM | 0.750 | 0.790 | 1.000 | 0.500 | 0.800 |
|  | SF-HTLM | 0.750 | 0.810 | 0.600 | 0.857 | 0.706 |
|  | E-HTLM | 0.750 | 0.830 | 0.500 | 1.000 | 0.667 |
|  | ACTL | 0.800 | 0.850 | 0.800 | 0.800 | 0.800 |
|  | TLCMI | 0.750 | 0.700 | 0.900 | 0.600 | 0.783 |
| A8 | **CHTLM** | 0.950 | 0.970 | 1.000 | 0.909 | 0.952 |
|  | I-CHTLM | 0.800 | 0.860 | 0.800 | 0.800 | 0.800 |
|  | SF-HTLM | 0.800 | 0.850 | 0.900 | 0.750 | 0.818 |
|  | E-HTLM | 0.850 | 0.850 | 0.900 | 0.818 | 0.857 |
|  | ACTL | 0.850 | 0.780 | 0.900 | 0.800 | 0.857 |
|  | TLCMI | 0.750 | 0.700 | 1.000 | 0.500 | 0.800 |
| Mean | **CHTLM** | 0.913 | 0.930 | 0.938 | 0.925 | 0.915 |
|  | I-CHTLM | 0.800 | 0.846 | 0.750 | 0.850 | 0.788 |
|  | SF-HTLM | 0.756 | 0.816 | 0.625 | 0.865 | 0.715 |
|  | E-HTLM | 0.781 | 0.820 | 0.675 | 0.867 | 0.750 |
|  | ACTL | 0.800 | 0.846 | 0.750 | 0.850 | 0.788 |
|  | TLCMI | 0.756 | 0.816 | 0.625 | 0.865 | 0.715 |

Dose H, Møller J S, Iversen H K, et al. An end-to-end deep learning approach to MI-EEG signal classification for BCIs[J]. Expert Systems with Applications, 2018, 114: 532-542.

Ma T, Wang S, Xia Y, et al. CNN-based classification of fNIRS signals in motor imagery BCI system[J]. Journal of Neural Engineering, 2021, 18(5): 056019.

Limpiti T, Seetanathum K, Sricom N, et al. Transfer Learning for Classifying Motor Imagery EEG: A Comparative Study[C]//2021 13th Biomedical Engineering International Conference (BMEiCON). IEEE, 2021: 1-5.
